# Engineering Adhesion of the Probiotic Strain *Escherichia coli* Nissle to the Fungal Pathogen *Candida albicans*

**DOI:** 10.1021/acssynbio.4c00466

**Published:** 2024-09-12

**Authors:** Alexandre Chamas, Carl-Magnus Svensson, Carla Maneira, Marta Sporniak, Marc Thilo Figge, Gerald Lackner

**Affiliations:** † Junior Research Group Synthetic Microbiology, Leibniz-Institute for Natural Product Research and Infection Biology, Jena 07745, Germany; ‡ Cluster of Excellence Balance of the Microverse, Friedrich Schiller University Jena, Jena 07743, Germany; § Applied Systems Biology, Leibniz-Institute for Natural Product Research and Infection Biology, Jena 07745, Germany; ∥ Institute of Microbiology, Faculty of Biological Sciences, Friedrich-Schiller University Jena, Jena 07743, Germany; ⊥ Chair of Biochemistry of Microorganisms, Faculty of Life Sciences: Food Nutrition and Health, University of Bayreuth, Bayreuth 95447, Germany

**Keywords:** live biotherapeutic product, probiotic, surface display, binding, *Candida albicans*, image analysis

## Abstract

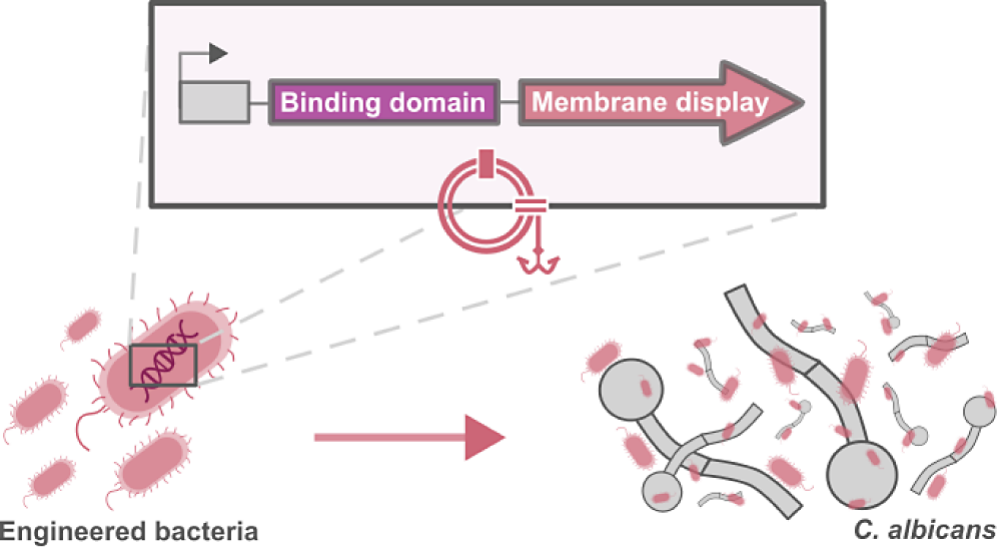

Engineering live biotherapeutic products against fungal pathogens such as *Candida albicans* has been suggested as a means to tackle the increasing threat of fungal infections and the development of resistance to classical antifungal treatments. One important challenge in the design of live therapeutics is to control their localization inside the human body. The specific binding capability to target organisms or tissues would greatly increase their effectiveness by increasing the local concentration of effector molecules at the site of infection. In this study, we utilized surface display of carbohydrate binding domains to enable the probiotic *E. coli* Nissle 1917 to adhere specifically to the pathogenic yeast *Candida albicans*. Binding was quantified using a newly developed method based on the automated analysis of microscopic images. In addition to a rationally selected chitin binding domain, a synthetic peptide of identical length but distinct sequence also conferred binding. Efficient binding was specific to fungal hyphae, the invasive form of *C. albicans*, while the yeast form, as well as abiotic cellulose and PET particles, was only weakly recognized.

## Introduction

Fungal infections are responsible for the death of 1.6 million people each year,^1^ and this figure is likely to increase due to the effect of global warming^2^ and the emergence of new fungal pathogens.^3^ This worrisome development prompted the World Health Organization to release a fungal priority pathogen list in order to alert the public about the risks of these infections and to encourage scientific progress toward their eradication.^4^ One of the member of this list, *Candida albicans*, is responsible for superficial infections but also for life-threatening invasive candidiasis.^5^ It exists in different morphotypes with the yeast form generally associated with commensalism and the hyphae form associated with virulence and damage.^6^ To treat a fungal infection, utilization of antifungals is generally the favorite measure, but the emergence of an increasing number of resistant strains threatens our capacity to efficiently handle these infections in the future.^7^ Therefore, in the global effort to treat fungal infections, the development of live biotherapeutic products (LBPs) represents a promising alternative to conventional antibiotics-based therapy. LBPs—also known as living therapeutics^8^ or engineered probiotics^9^—are commensal members of our microbiome, modified to have an enhanced therapeutic value. They can be genetically modified to produce effector compounds or sense a particular physiological cue,^10,11^ and they can also be surface-modified to better cope with hostile milieu and reach their site of action.^12^

In the past years, several LBPs have been developed that can sense, kill, or attenuate the virulence of diverse pathogens like *Pseudomonas aeruginosa*,^13^*Salmonella enterica,*^14^ or *Candida albicans*.^15^ In one of the first attempts to fight fungal infections with therapeutic microbes, the bacteria *Escherichia coli* were utilized to produce the *Candida*-specific antivirulence compound BDSF in vivo.^15^ The heterologous synthesis of BDSF was shown to efficiently block yeast-to-hyphae morphogenesis in vitro and drastically reduce yeast pathogenicity in model experiments. This work as well as other developments of therapeutic microbes is paving the way for the establishment of a new branch of therapeutic agents against fungal infections.^9,16,17^

BDSF is only one of the several chemical small molecules, proteins, or small oligopeptides shown to either limit virulence, inhibit growth, or kill *C. albicans*.^18,19^ Because a large proportion of these molecules are natural products, many of them could potentially be produced by therapeutic microbes, especially in the light of the large panel of chassis organisms available and the recent advances in synthetic biology.^20,21^ However, one of the major challenges of this approach remains the reach of sufficiently high concentrations to observe an antifungal effect on the target. Achieving high yields of heterologously produced compounds often represents a heavy burden for therapeutic microorganisms as chassis strains are generally not selected for efficient recombinant protein production but rather for good adaptation to the host environment and overall safety.^22,23^ In comparison to highly modified laboratory strains, therapeutic microbe chassis often shows lower production titers for heterologous compounds and lower tolerance to genetic engineering.^24^ Additionally, the production of high concentrations of antifungal compounds in vivo raises substantial risks during treatment as high doses of effector molecules can cause adverse effects on host cells or on members of the microbiome. Another strategy for the supply of high amounts of antifungal compounds via therapeutic microbes could be the supply of large quantities of the designated microbe. Nevertheless, this could lead to the destabilization of the host microbiome and consequently present health risks for the patient.^25^

In response to these limitations and to fully exploit the large scope of antifungal molecules, we aim to add another feature to therapeutic microbes: the possibility of specifically adhering to specifically adhere to its target. Adhesion would allow the LBP to remain in the proximate vicinity of a pathogen for a long period and potentially increase the local concentration of the effector molecule at the target.

*E. coli* was already engineered for adhesion to diverse abiotic surfaces and metals by means of surface display of catecholamines^26^ as well as for binding to tumors, with the display of synthetic adhesins^27^ or histone-like protein A.^28^ In the latter case, the well-known probiotic *E. coli* Nissle 1917 (EcN) was used as chassis. One of the few examples where *E. coli* was designed to adhere to another microorganism was achieved by displaying lectins to bind to cyanobacteria.^29^ Here, our goal was to develop an adherent version of EcN that targets the pathogen *C. albicans*.

Even though various display systems exist, their application in *E. coli* is challenging.^30,31^ Popular display systems are the ice nucleation protein (INP, either the whole protein or the N-terminus),^32,33^ the adhesin involved in diffuse adherence (AIDA),^34,35^ or more recently the fimbriae protein FimH.^36^ However, the nature of the passenger protein, the strength of the promoter-driving protein expression, and the genetic background of the strain have a great influence on display success, and no “fit-for-all” system has been established yet. It is important to note that most of the work on surface display was performed with common lab strains of *E. coli* instead of the probiotic strain EcN. The variability of membrane display success is even higher in EcN because this strain possesses a different outer membrane composition than the common lab strains BL21 (DE3) or MG1655 with three different types of fimbriae and flagella.^37^ Moreover, the establishment of robust promoters, terminators, and induction systems has been described only recently for EcN.^38,39^

*C. albicans* is naturally recognized in the human body by a wide range of receptors binding to the three carbohydrates present in the fungus cell wall.^40,41^ These three carbohydrates are chitin, an oligosaccharide of *N*-acetylglucosamine, β-glucans (both β-(1,3) glucans and β-(1,6) glucans), and mannans (as part of protein–mannan complexes). Their proportion in the total content of the cell wall depends on the physiological state but equals roughly 1–10% chitin, 20–60% glucans, and the remainder mannoproteins.^42,43^ Hyphae or pseudo-hyphae generally possess more chitin in their cell wall than the yeast form but display a less amount of mannans.^43^ This cell wall composition is not specific to *C. albicans,* and other yeasts like *S. cerevisiae* present a very similar cell wall with minor changes in the percentage of each carbohydrate. In contrast, other common fungal pathogens present strikingly different cell walls containing α-glucan (*Histoplasma capsulatum*), melanin (conidium cell wall of *Aspergillus fumigatus*), or galactomannans (*A. fumigatus* and *Cryptococcus neoformans*).^44^ The adhesion between *C. albicans* and the Gram-positive bacterium *Staphylococcus aureus* has been documented. In this case, binding occurs through an interaction of the hyphal-specific Als3 fungal adhesin with an unknown bacterial partner.^45^ The Gram-negative *Pseudomonas aeruginosa* is known for its ability to kill *C. albicans*, and it was shown that adhesion of the bacteria to glycan moieties of the fungus hyphae was involved in this process.^46^ This binding was reported to be mediated by the bacterial chitin binding protein CbpD.^47^

In this study, we displayed membrane proteins with affinity for the components of the *C. albicans* cell wall on the surface of EcN to achieve adhesion to the fungus. To this end, we selected passenger proteins with carbohydrate binding properties and investigated their display efficiency. Our best candidates were then incubated with hyphae of *C. albicans*, and the adhesion between the two organisms was quantitatively assessed.

## Materials and Methods

### Strains, Media, and Chemicals

*E. coli* strain Stellar (Clontech Laboratories, Inc., Mountain View, USA) was utilized for plasmid cloning. *E. coli* strains BL21 (DE3) (New England BioLabs, Ipswich, USA) and Nissle 1917 (DSM 6601, Jena Microbial Resource Collection, Jena, Germany) were utilized for the membrane display of carbohydrate binding domains. Partial deletion of the colibactin-producing cluster in *E. coli* Nissle 1917 was performed by the help of the no scar-mediated deletion protocol,^48^ thus creating the strain *E. coli* Nissle 1917: *ΔclbK-clbQ* which was used for all experiments (Figure S1). *C. albicans* strain SC5314 (ATCC 28 367) was obtained from the Jena Microbial Resource Collection (Jena, Germany). *E. coli* strains were cultivated in the Luria–Bertani medium (LB) supplemented with 100 μg/mL kanamycin (Carl Roth GmbH + Co KG, Karlsruhe, Germany). *C. albicans* SC5314 was cultivated in the Yeast Peptone Dextrose medium (YPD) at 37 °C. Yeast-to-hyphae transition was initiated by performing a 1:50 dilution in the KBM medium (Lonza Ltd., Basel, Switzerland) and incubating this culture overnight at 37 °C under vigorous shaking. Unless indicated otherwise, all chemicals were obtained from Merck KGaA (Darmstadt, Germany).

### Cloning and Transformations

Genetic parts utilized in this study were ordered as gene fragments or as oligonucleotides from Eurofins Genomics (Ebersberg, Germany) and are listed in Table S1. All PCR reactions were performed with Q5 polymerase (New England BioLabs, Ipswich, USA), and all plasmids, based on pET-28 a (+) (Merck KGaA, Darmstadt, Germany) backbone, were built with Hifi homology assembly (New England BioLabs, Ipswich, USA), following manufacturer protocols. Sequences of the expression cassettes were verified via Sanger sequencing (Eurofins Genomics, Ebersberg, Germany) with the primers listed in Table S2.

All *E. coli* strains were transformed by heat-shock following the Stellar competent cell transformation protocol (Clontech Laboratories, Inc., Mountain View, USA) and selected on LB + kanamycin plates.

### Promoter Test

A colony of *E. coli* Nissle 1917: *ΔclbK-clbQ* freshly transformed with pOK1 or pOK2 was incubated overnight in 3 mL LB + kanamycin under vigorous shaking. This preculture was diluted 1:100 in 15 mL of fresh LB + kanamycin and incubated at 37 °C and 210 rpm for 18 h in triplicate. 1 mL of this culture was centrifuged at 13000 rpm, and the pellet was washed with 1 mL of phosphate buffer saline (PBS, pH = 7.4) and resuspended in 1 mL of PBS. 200 μL of this suspension was added to a 96-well black microtiter plate with μclear® bottom (Greiner Bio-One GmbH, Frickenhausen, Germany) for the measurement of OD at 700 nm and mKate2 fluorescence (λ_excitation_ = 588 nm, λ_emission_ = 634 nm) in a fluorescence microplate reader (Clariostar plus, BMG Labtech, Ortenberg, Germany). After subtraction of the blank, the fluorescence values were normalized by dividing them by their respective OD_700_.

### Microbial Cultures

A colony of *E. coli* Nissle 1917: *ΔclbK-clbQ* or BL21(DE3) freshly transformed with the relevant plasmid was incubated overnight in 3 mL LB + kanamycin under vigorous shaking. This preculture was diluted 1:100 in 15 mL of fresh LB + kanamycin and incubated at 37 °C and 210 rpm for 18 h. 1 mL of this culture was centrifuged at 13000 rpm, and the pellet was washed with 1 mL of PBS and resuspended in 1 mL of PBS. After measurement of OD at 700 nm, all cultures were diluted with PBS to obtain 1 mL at OD_700_ = 0.8 for SDS-PAGE and Western blot or OD = 2 for all other experiments.

*C. albicans* SC5314 from a freshly streaked plate was grown overnight in 3 mL of YPD at 37 °C under vigorous shaking. For the growth of *C. albicans* hyphae, 80 μL of this preculture was added to 6 mL of the KBM medium and incubated overnight at 37 °C and 210 rpm to induce yeast-to-hyphae transition. 5 mL of hyphae was then centrifuged at 13000 rpm for 3 min, and the supernatant was carefully removed. After addition of 5 mL sterile PBS, the suspension was centrifuged again at 13000 rpm for 3 min, and the supernatant was carefully removed. Finally, the washed hyphae were resuspended in 5 mL of sterile PBS. For the growth of *C. albicans* as yeast, 80 μL of the preculture was added to 4 mL of YPD and incubated overnight at 37 °C and 210 rpm. 2 mL of the yeast culture was centrifuged at 13000 rpm for 3 min, and the supernatant was removed. After addition of 2 mL sterile PBS, the suspension was centrifuged again at 13000 rpm for 3 min, and the supernatant was removed. Finally, the yeast pellet was resuspended in 1 mL sterile PBS.

### SDS-PAGE and Western Blot

In a microcentrifuge tube, 50 μL of the desired bacterial suspension was mixed with 10 μL of 6× SDS-PAGE loading dye containing 2-mercaptoethanol, and tubes were incubated for 10 min at 95 °C. 25 μL of samples was added to the well of a 4–12% mPAGE® bis-tris precast gel (Merck KGaA, Darmstadt, Germany) and submitted to gel electrophoresis for 26 min at 180 V. PageRuler Prestained Protein Ladder (Thermo Fischer Scientific, Inc., Waltham, USA) was utilized in the first well of the gel.

After electrophoresis, the SDS gel was submitted to Western blot for the detection of FLAG-tagged-proteins. For that, all proteins were transferred overnight to a PVDF membrane (Thermo Fischer Scientific, Inc., Waltham, USA) with the help of a mini Trans-Blot Electrophoretic Transfer Cell (Bio-Rad Laboratories, Inc, Hercules, USA) following manufacturer’s recommendations. The membrane was then washed three times with 10 mL PBS + 0.03% Tween 20 (PBS-T) for 5 min and blocked for 1 h at room temperature under agitation with 40 mL of 3% milk in PBS-T. After three washes with 10 mL PBS-T for 5 min, the membrane was incubated with a 1:2000 dilution of DYKDDDDK Antibody, HRP, REAfinity (Miltenyi Biotec B.V. & Co. KG, Bergisch Gladbach, Germany), in PBS-T at room temperature for 1 h under agitation. The membrane was washed again three times with 10 mL PBS-T for 5 min, and detection of HRP activity was performed by using Pierce SuperSignal West Pico PLUS Chemiluminescent Substrate (Thermo Fischer Scientific, Inc., Waltham, USA) following the manufacturer protocol. Observation of chemiluminescence was achieved in the Azure 280 gel documentation system (Azure Biosystems, Dublin, USA).

### Antibody Labeling of Membrane Proteins

Two tubes of the desired bacterial suspension were centrifuged at 13000 rpm for 1 min, the supernatant was discarded, and the pellet was resuspended in 100 μL PBS + 4% formaldehyde. Tubes were incubated for 15 min at room temperature and then washed with 500 μL PBS. After centrifugation and removal of the supernatant, one of the two duplicates was resuspended in 50 μL PBS (no antibody control), and the other was resuspended in 50 μL DYKDDDDK antibody, FITC (Miltenyi Biotec B.V. & Co. KG, Bergisch Gladbach, Germany), diluted 1:50 in PBS (sample with antibody). All tubes were incubated 45 min at 4 °C in the dark and then centrifuged at 13000 rpm for 3 min. After careful resuspension with 1 mL PBS, the tubes were centrifuged again at 13 000 rpm for 3 min, and the supernatant was discarded. Finally, 100 μL of PBS was added to each tube, and the resuspended pellets were added to a black microtiter plate with μclear® bottom for measurement of OD at 700 nm and FITC fluorescence (λ_excitation_ = 483 nm, λ_emission_ = 530 nm) in a microtiter plate reader. For each sample, three fluorescence measurements were performed (technical replicates). The fluorescence values were normalized by dividing them by their respective OD_700_ values, and the values of the three replicates were averaged. The same experiment was performed three times on different days (biological replicates) for the EcN transformants, and these replicates were used for data representation and statistical tests. For BL21(DE3), the results of the technical replicates for one experiment are shown.

### Binding Assays with *C. albicans* Hyphae

In a sterile microcentrifuge tube, 250 μL sterile PBS was mixed with 200 μL of washed hyphae and 50 μL of each diluted *E. coli* strain. All tubes were incubated for 18 h without shaking at 37 °C and then passed through a 30 μm nylon cell strainer (pluriSelect Life Science, Leipzig, Germany). Each strainer was washed with 3 mL of sterile PBS and subsequently flipped. 200 μL of sterile PBS was added to the inverted strainer to recover the hyphae in a new falcon. 6 μL of the hyphae was mounted on a microscope slide and observed with an EC Plan-Neofluar 40*x*/0.75 objective of an Axiolab 5 microscope (Carl Zeiss Microscopy Deutschland GmbH, Oberkochen, Germany). For each strain, three positions on the slide were randomly chosen, and for each position, one bright-field and one fluorescence image were taken with an Axiocam 202 (Carl Zeiss Microscopy Deutschland GmbH, Oberkochen, Germany) camera (0.83× magnification, final magnification 33.2×). Fluorescence images were taken with the help of a 590 nm LED light source for excitation and a 92 HE LED filter set (Carl Zeiss Microscopy Deutschland GmbH, Oberkochen, Germany) for observation. Merging of channels and image export were performed with the help of ZEN blue edition 3.2 software (Carl Zeiss Microscopy Deutschland GmbH, Oberkochen, Germany).

### Binding Assays with *C. albicans* as Yeast

In a sterile microcentrifuge tube, 250 μL sterile PBS was mixed with 100 μL of washed *C. albicans* as yeast and 10 μL of each diluted *E. coli* strain. All tubes were incubated for 18 h without being shaken at 37 °C. 6 μL of each mixture was mounted on a microscope slide and observed with an Axiolab 5 microscope as described previously.

### Binding Assays with Abiotic Surfaces

Chitin bead suspension was prepared by the following procedure: 1 mL of chitin resin stored in 20% ethanol (New England BioLabs, Ipswich, USA) was centrifuged at 13000 rpm for 3 min, and 600 μL of the ethanol-containing supernatant was carefully removed. Then, 600 μL of sterile PBS was added, the suspension was centrifuged at 13000 rpm for 3 min, and 600 μL of the supernatant was carefully removed. This operation was repeated five times to ensure that almost all ethanol was washed away from the beads. Cellulose and PET suspensions were prepared by adding, respectively, 10 mg of cellulose (Carl Roth GmbH + Co KG, Karlsruhe, Germany) or 10 mg of semicrystalline polyethylene terephthalate powder of 300 μm (Goodfellow, Huntington, UK) to 1 mL of sterile PBS.

In a sterile microcentrifuge tube, 250 μL sterile PBS was mixed with 100 μL of either cellulose or PET suspension and 10 μL of each diluted *E. coli* strain. For the binding assay with chitin beads, the mixture was consisting in 250 μL sterile PBS, 100 μL of beads, and 50 μL of each diluted *E. coli* strain. All tubes were incubated for 18 h without shaking at 37 °C. 6 μL of each mixture was mounted on a microscope slide observed with an Axiolab 5 microscope as described previously.

### Quantification of Binding

Determination of the amount of bacteria bound to *C. albicans* hyphae was performed by the ImageJ-based visual batch processing tool JIPipe.^49,50^ In the hyphae channel, Gaussian smoothing with a radius of 3 pixels to suppress noise and a Sobel edge detector to find the contour of the hyphae were used. To ensure that the contour is connected and closed, closing with lines directed in the orientations 0, 45, 90, and 135 degrees was utilized. The subsequent thresholding was performed using ImageJ’s default auto threshold, and then, opening with the same lines as closing was applied to the binary image. The bacteria channel was repeated at several focal planes to ensure that all bacteria were visible. After background subtraction with a rolling ball algorithm with a radius of 50 pixels and histogram-based contrast enhancement with 0.05% saturated pixels, a max projection across the focus planes and manual thresholding at intensity 20 were performed. After segmenting both channels, the percentage of hyphae area that was covered by bacteria was calculated. The JIPipe pipeline is available at https://github.com/applied-systems-biology/Nissle-binding.

### Protein Structure Predictions

Protein structure predictions were performed by submitting the respective protein sequences to ColabFold v1.5.3: AlphaFold2 tool.^51^ For each protein, highest-ranked prediction was visualized with Mol* viewer.^52^ The protein structure was colored according to the pLDDT value which describes the uncertainty/disorder of the prediction for each residue. Blue coloring indicates a pLDDT > 70 and therefore modeling with high accuracy, whereas red coloring indicates a pLDDT < 30 which accounts for low accuracy modeling.

### Statistical Tests

For all charts presented, the average value of the different replicates is indicated, with the error bars describing the standard deviation. All statistical tests were performed with SigmaPlot v15 (Inpixon, Palo Alto, USA), and the test utilized is indicated in the legend of each figure.

## Results and Discussion

### Cloning of Membrane-Displayed Proteins Using the AIDA System

**Figure 1:**
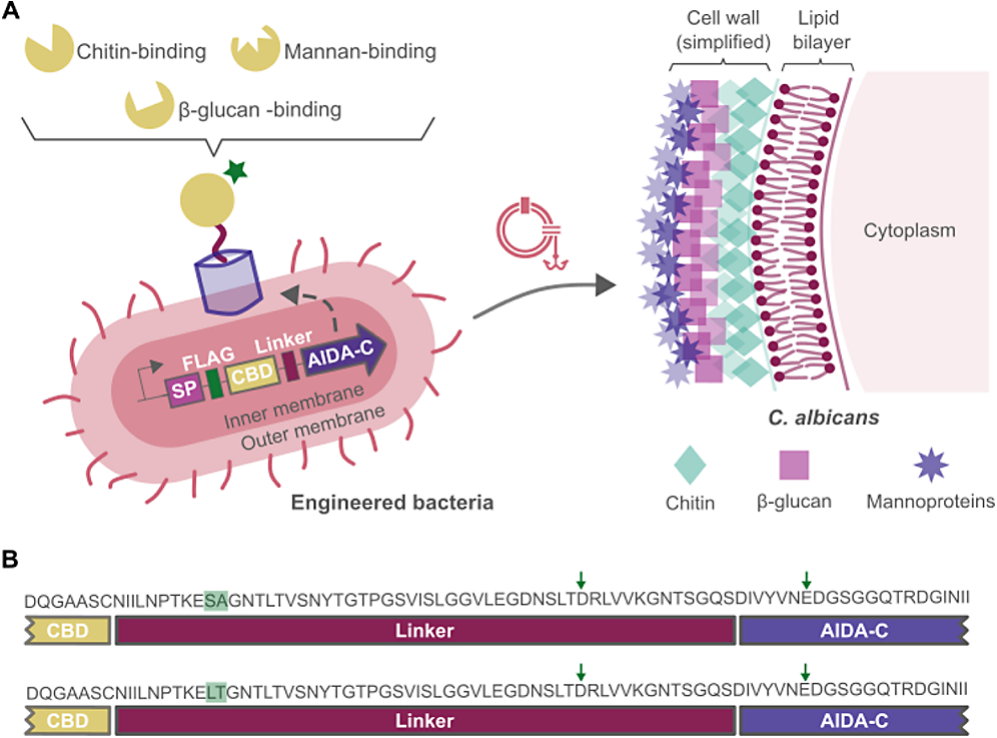
(A) Schematic representation of the engineered EcN for the AIDA-mediated membrane display of proteins and of the cell wall of *C. albicans*. SP: signal peptide; CBD: carbohydrate binding domain. The green star represents the FLAG tag. (B) Protein sequences of the two different linkers used, the native linker (top) and the noncleavable linker (bottom). Differences in amino acids composition are highlighted in green. Amino acids responsible for the autoproteolytic cleavage of the fusion protein are indicated with arrows.

Despite a large choice of strategies available, membrane protein display in *E. coli* remains challenging. Since preliminary experiments showed that the INP-display system was leading to the accumulation of misfolded proteins in inclusion bodies in both the *E. coli* BL21 and Nissle strains, we focused on the utilization of the AIDA display system. The AIDA system necessitates the fusion of the passenger protein, in our case, a carbohydrate-binding domain with affinity for *C. albicans*, to the C-terminal part of the native AIDA protein. For this purpose, we designed plasmids—based on a pET-28 (+) a backbone—containing a constitutive synthetic promoter, an N-terminal membrane signal sequence, a FLAG tag, a carbohydrate binding domain (CBD), and a linker fused to the C-terminal carrier part of the AIDA protein. Additionally, all plasmids contain an expression cassette for the constitutive expression of the mKate2 fluorescent protein. The general architecture of the expression cassettes can be seen in Figure 1A.

Utilization of a constitutive promoter was preferred to ensure the correct expression of the constructs in the majority of *E. coli* strains, independently of repressor/inducer systems. These regulators may be absent from certain genotypes and especially from the probiotic strain EcN. Promoters of the Anderson collection are small and characterized genetic parts whose sequences are freely available.^53^ The strength of two different promoters of the Anderson collection (low-strength BBa_J23114 and high-strength BBa_J23100) in EcN was assessed by placing them upstream of a reporter gene encoding red fluorescent protein mKate2. Fluorescence intensity was measured, allowing us to compare the promoter strength in EcN to the strength calculated in the original description. Interestingly, the low-strength BBa_J23114 promoter diplayed 30% of the activity of BBa_J23100 in EcN, while reaching 10% in the original description of the promoter (Figure S2). Even though selecting the high-strength promoter BBa_J23100 seemed favorable in order to achieve the strongest possible protein expression, cloning the strong promoter in the construct described in Figure 1A led to very few colonies after transformation, all bearing frame-shift mutations in the membrane protein sequence. This indicates a probable toxicity of the heterologous protein at high concentration as the cloning of the lower-strength promoter BBa_J23114 did not cause these issues. Therefore, the BBa_J23114 promoter was selected for all the following experiments.

An extensive literature search was performed to find naturally occurring proteins binding to chitin, β-glucan, or mannan. From these binding proteins, three carbohydrate binding domains were selected for surface display. Decisive parameters to guide the selection were (I) binding activity as monomer, (II) successful heterologous production in *E. coli*, and (III) a relatively small carbohydrate-binding domain or documented binding to *C. albicans*. A description of all of the passenger proteins used in our setup with their corresponding sequence can be seen in Table 1. The bacterial chitinase 92 protein was already heterologously produced as a whole protein on the surface of *E. coli* BL21 with the help of the INP-display system.^54^ Chitinase 92 has two chitin binding domains at its C-terminus, and both of them were utilized to form carbohydrate binding domains. Actinohivin is a lectin originating from the actinomycete *Longispora albida* with a carbohydrate binding module family 13 domain with affinity for mannose.^55^ Dectin-2 is a mammalian receptor involved in the recognition of several fungal pathogens and has an affinity for mannose.^56^ It is the only domain of eukaryotic origin. Additionally, we included synthetic peptide SP1, which has the same sequence length as the chitinase 92 binding domain. Its sequence contains a proportion of amino acids mimicking the average composition of amino acids in all proteins present in the UniProtKB database generated by the Randseq tool from Expasy.^57^ This peptide was selected because it is documented that carbohydrate binding partially relies on hydrophobic interactions involving aromatic amino acids, and we were therefore expecting a random sequence to also show some level of attachment.^58^ As a control, synthetic peptide SP2 was designed. It possesses the same sequence as SP1 but with all aromatic amino acids exchanged for nonaromatic ones. Additionally, some hydrophobic amino acids were exchanged for hydrophilic residues to lower the overall hydrophobicity of the carbohydrate binding domain of SP2.

**Table 1:** Description of the Strains Used in This Study

Protein	UNIPROT accession number	Binding domain sequence	Size after fusion with C-AIDA (kDa)	% aromatic amino acids in binding domain	Affinity	Origin	Strain
Actinohivin	Q9KWN0	ASVTIRNAQTGRLLDSNYNGNVYTLPANGGNYQRWTGPGDGTVRNAQTGRCLDSNYDGAVYTLPCNGGSYQKWLFYSNGYIQNVETGRVLDSNYNGNVYTLPANGGNYQKWYTG	62.026	14	Mannan	*Longispora albida* gen. nov., sp. nov	EcN/act
Chitinase 92	Q9F9Q8	HPAWSAGTVYNTNDKVSHKQLVWQAKYWTQGNEPSRTADQWKLVSQVQLGWDAGVVYNGGDVTSHNGRKWKAQYWTKGDEPGKAAVWVDQGAASCN	60.205	12.5	Chitin	*A. hydrophila*	EcN/chi
Dectin-2 (CLEC6A)	Q9JKF4	RRLYELHTYHSSLTCFSEGTMVSEKMWGCCPNHWKSFGSSCYLISTKENFWSTSEQNCVQMGAHLVVINTEAEQNFITQQLNESLSYFLGLSDPQGNGKWQWIDDTPFSQNVRFWHPHEPNLPEERCVSIVYWNPSKWGWNDVFCDSKHNSICEMKKIYL	68.315	14.4	Mannan	*M. musculus*	EcN/dec
Synthetic peptide_01	-	IKIEFTSREVPWNSKLDGYLDDGATRLFAYQQNHPVAAVLSTRIMYGPISRQIARADEALHTFSNPLILVADKKSLVPVEKGTFMLEGNQCGVEGK	60.166	8.3	-	-	EcN/SP1
Synthetic peptide_02	-	AKAESTSREVPGNSKLDGRLDDGATRLSAGQQNHPVAAVLSTRAMGGPNSRQDARADEALHTGSNPGSGVADKKSLVPVEKGTAMLEGNQCGVEGK	59.269	0	-	-	EcN/SP2

The AIDA-mediated display of proteins on the surface of *E. coli* was performed via fusion of the passenger protein to the C-terminal translocator domain of the AIDA protein. As a member of the type V secretion systems, the system performs two steps to efficiently secrete or display proteins in the extracellular space.^59^ First, an N-terminal signal sequence allows the protein to cross the inner membrane, and this signal tag is cleaved inside the periplasm. Next, the C-terminal translocator domain is embedded in the outer membrane, thus allowing secretion of the passenger part into the milieu. Interestingly, the secreted part of the protein is then cut from the translocator domain but remains tightly associated with the membrane in a still poorly understood manner.^60,61^ It was found previously that two amino acids in a linker region directly upstream of the translocator domain have a great influence on the cleavage efficiency. Replacing the native serine–alanine motif by leucine–threonine prevented this cleavage.^62^ Because our aim was the display of proteins rather than secretion, we opted for two designs: one with the native linker upstream of the translocator domain, which should allow for cleavage and tight association (referred to in this study as a native linker) and one design with the modified version containing the leucine–threonine motif (referred to as a noncleavable linker). A comparison of the two linker regions at the sequence level can be seen in Figure 1B, where the two amino acids responsible for the cleavage, an aspartic residue and a glutamic acid residue, are also indicated.

All membrane protein expression cassettes were inserted in a modified pET28-a-(+) vector containing another expression cassette for the production of red fluorescent protein mKate2 under the control of constitutive promoter P_CAT_. This modification was added to allow the quick identification of transformed strains and to track the cells in subsequent assays. Finally, a negative control strain (EcN/control) was constructed by transforming EcN with a plasmid containing only the expression cassette for mKate2 production.

As an expression host, we developed an EcN strain containing a partial deletion of the colibactin (*clb*) gene cluster. Colibactin is a genotoxic compound which renders this probiotic potentially cancerogenic for humans.^63^ Successful deletion of genes *clbK-clbQ* of the colibactin synthesis cluster was confirmed by sequencing. Strains were named according to the fusion constructs that they contain (Table 1).

### Production and Display of Membrane Proteins

**Figure 2:**
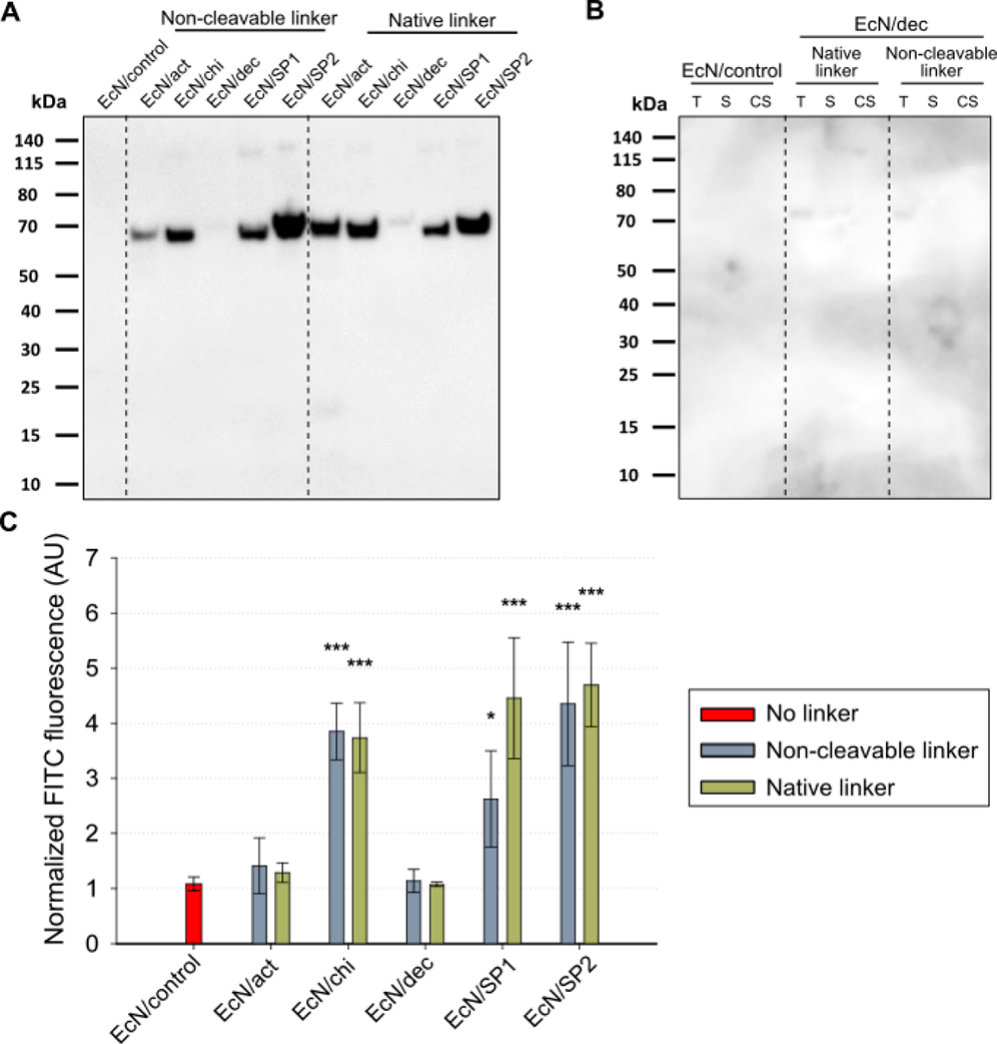
(A) Western blot analysis for the detection of membrane fusion protein in the total protein content of the indicated strains. (B) Western blot analysis for the detection of membrane fusion protein in different fractions of EcN/dec. T: total protein content, S: medium supernatant, and CS: concentrated medium supernatant. (C) Immunolabeling of the indicated strains for the detection of membrane proteins. Measurement of fluorescence after incubation of cells with an anti-FLAG antibody conjugated with FITC was divided by the fluorescence of the same strains without antibody incubation. Error bars represent the standard deviation of three biological replicates. The statistical test performed was a one-way ANOVA with multiple comparisons versus EcN/control (Holm–Sidak method): *p < 0.05, ^**^p < 0.01, and ^***^p < 0.001.

Production of the different membrane proteins was assessed for the modified EcN strains by Western blot after incubation with an anti-FLAG antibody (Figure 2A). The theoretical molecular weights of the different fusion proteins after cleavage of the N-terminal signal peptide can be found in Table 1. Strain EcN/control did not exhibit any band, an expected result, as the plasmid in this strain does not express any AIDA-fused protein and therefore no FLAG tag. For both the native and noncleavable linkers, a strong band showing the presence of the tagged protein can be observed between 50 and 70 kDa for EcN/act, EcN/chi, EcN/SP1, and EcN/SP2. For EcN/dec, only a very faint band can be observed at a higher molecular mass than the other fusion proteins, in accordance with the larger size of the dectin-2 passenger domain. Additionally, we could observe faint bands for EcN/chi, EcN/SP1, and EcN/SP2 between 115 and 140 kDa for both linkers, which may be attributed to the formation of dimers. Finally, this Western blot also revealed the presence of a faint band between 15 and 25 kDa for EcN/act with the native linker, a result hinting that for this construct, the AIDA-2 cleavage of the membrane protein does occur.

The faint band observed in the case of the dectin-2-AIDA fusion protein may be the result of poor protein expression, increased degradation, or secretion to the extracellular milieu. In its native form, the AIDA protein is cleaved and remains associated with the cell membrane.^59^ One could reason that, in the case of EcN/dec, cleavage occurs, but the cleaved fragment does not remain associated with the membrane; nevertheless, the faint band was also observed with the noncleavable linker. To further investigate this issue, the culture supernatant of EcN/dec with the native or noncleavable linker was concentrated and subjected to SDS-PAGE and Western blot detection. No band could be observed in the supernatant at a size corresponding to the cleaved product (20 kDa), the only visible bands being again faint signals in the total protein samples (Figure 2B). This strengthens the hypothesis that the dectin-2-AIDA fusion product is not produced efficiently in EcN. Dectin-2 is the only protein of mammalian origin which has been selected for display, and the carbohydrate binding domain of this protein contains eight cysteines and consequently forms four disulfide bonds. Disulfide bond formation is generally difficult in *E. coli,* and the absence of these linkages can have a negative impact on protein stability and solubility besides activity.^64^ Production of the carbohydrate binding domain dectin-2 in *E. coli* BL21 has been documented before, resulting in proteins forming inclusion bodies and therefore highlighting the poor solubility of dectin-2 in *E. coli*.^65^ In our setup, this poor solubility may lead to the breakdown of the fusion protein through the bacterial protein degradation machinery and could explain the very faint band observed in the Western blot.

Display of the different carbohydrate binding proteins on the membrane of EcN was tested by immunolabeling with an FITC-conjugated antibody directed against the FLAG tag. This antibody can bind to proteins only at the surface of EcN and not penetrate inside the cell. The results of whole-cell immunolabelling can be seen in Figure 2C. While strain EcN/control does not show any fluorescence signal, significant fluorescence was observed for the strains EcN/chi, EcN/SP1, and EcN/SP2—with both the native and noncleavable linkers—confirming the presence of correctly folded fusion proteins on the surface of these strains. In accordance with the Western blot results, EcN/dec did not display fluorescence with either linker. More surprisingly, EcN/act also did not exhibit significant labeling with any of the two linkers, although good protein expression was observed by Western blot. To confirm that the immunolabeling signal obtained was not due to unspecific binding to the AIDA translocator domain or the diverse carbohydrate binding domain, additional strains containing the identical expression cassette but lacking a FLAG tag were constructed. It showed no fluorescence signal (Figure S3).

To check if the strain background could explain the lack of membrane display for EcN/act, plasmids encoding the fusion protein were used to transform *E. coli* BL21, and the results of antibody labeling can be seen in Figure S4. Overall, similar results as for EcN can be seen in BL21 with *E. coli* BL21/chi, *E. coli* BL21/SP1, and *E. coli* BL21/SP2 showing good display, while *E. coli* BL21/act and *E. coli* BL21/dec did not exhibit any significant labeling.

This experiment confirms the successful membrane display of proteins on EcN utilizing AIDA-fusion, a strategy described only in a few publications.^66−68^ One of them used the AIDA-fusion strategy for the display of coronavirus spike proteins on the bacterial membrane, highlighting the advantages of this particular display system for EcN applications.^66^ Our experiments show that protein transport to the membrane and efficient display are highly dependent on the nature of the fused domain to the C-terminal AIDA protein. Although the actinohivin-AIDA fusion is well-expressed in EcN, as shown by the Western blot experiments, no protein could be detected in the membrane through immunolabelling. This can be the result of a problem during protein transport, leading to a wrong localization inside the cell or by difficult accessibility of the antibody to the FLAG tag in nondenaturing conditions. To examine if the protein structure of the actinohivin-AIDA fusion could help explain these results, a structure simulation of all fusion proteins designed in this study (Figure S5) was performed using Alphafold2.^51,69^ For each structure, the backbone was colored according to the pLDDT structure prediction score, and the N-terminus containing the FLAG tag was indicated by a green arrow. No obvious steric hindrance that could prevent antibody binding was observed in the case of the chi-AIDA fusion protein. It is necessary to consider that the N-terminal region of the simulated structures has a lower prediction score than the rest of the protein, and one cannot rule out the possibility that the FLAG tag is not accessible to the anti-FLAG antibody in native conditions. Nevertheless, simulations suggested that the wrong localization of the produced protein inside the cells may be the more plausible explanation for the lack of antibody labeling.

### Binding to *C. albicans* Hyphae and Image Analysis for Binding Quantification

**Figure 3:**
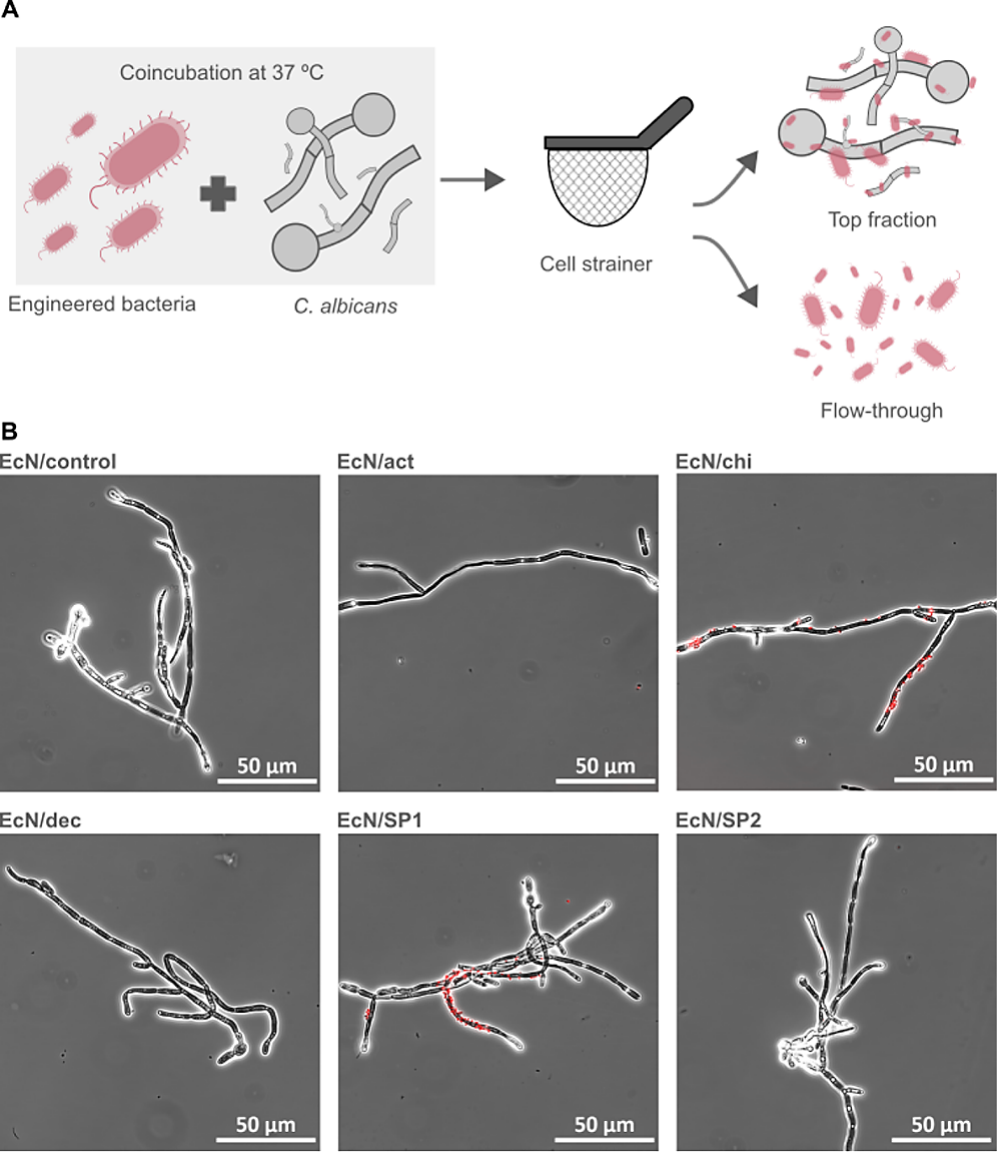
(A) Schematic representation of the hyphae binding assay setup. Red cells represent engineered EcN, and gray filaments represent *C. albicans* hyphae. (B) Representative microscope images of the attachment between *C. albicans* hyphae and the indicated strains. Images are a merger of the bright-field and red fluorescence channels.

To test if the different strains can effectively bind to the pathogenic dimorphic yeast *C. albicans*, a coincubation assay was designed (Figure 3A). Because Western blot and immunolabeling experiments did not show any difference in protein display for the strains with the native or noncleavable linker, only the strains producing fusion proteins with a noncleavable linker were used for the further experiments. EcN strains and *C. albicans* hyphae were cultivated separately and mixed together, and this mixture was incubated at 37 °C for 18 h. Hyphae with *E. coli* cells attached were separated from free-floating bacteria by passing through a 30 μm cell strainer. With this device, the 1–5 μm sized bacteria can pass through the mesh, while the much larger hyphae remain on the top. The top fraction was observed under the microscope (Figure 3B). The presence of bacteria attached to the hyphae was observed when EcN/chi and EcN/SP1 were mixed with the hyphae, whereas all other bacterial strains did not show visible binding.

**Figure 4:**
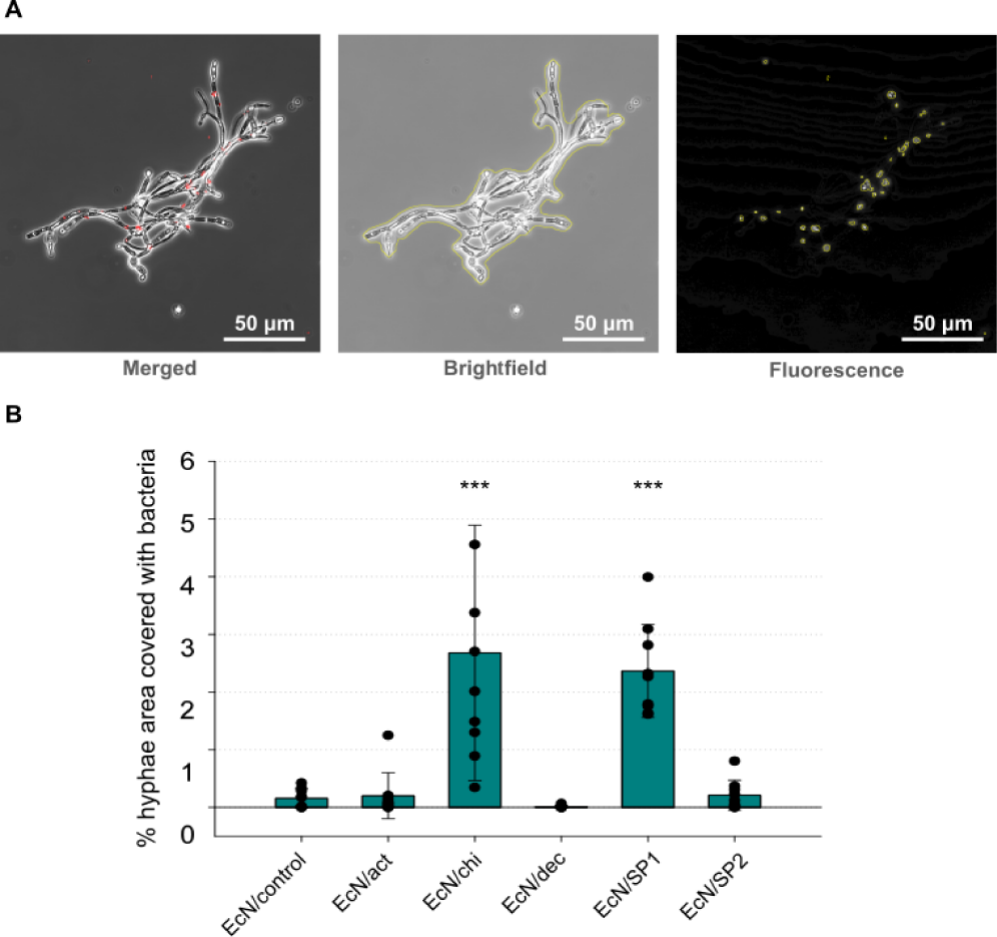
(A) Example of segmentation performed with the JIPipe image tool. The two channels (bright-field, in the middle and fluorescence, on the right) of the final microscope image (on the left) were used for the determination of hyphae area and bacteria area, respectively. The yellow line depicts the boundaries of hyphae and bacteria as determined by JIPipe. (B) Percent hyphae covered with bacteria after the hyphae binding assay for the indicated strains after incubation for 18 h. Dots show the values of nine replicates, and bars show their mean with error bars representing the standard deviation. The statistical test performed was a one-way ANOVA with multiple comparisons versus EcN/control (Holm–Sidak method): *p < 0.05, ^**^p < 0.01, and ^***^p < 0.001.

Because visual observation of microscopic images does not allow precise quantification, we developed an automated image analysis pipeline implemented in JIPipe^50^ to quantify the binding of EcN to *C. albicans* hyphae. The first step in the analysis workflow was to provide a microscopic image of an EcN/hyphae mixture containing both the bright-field and the red fluorescence channel as input for the analysis tool (Figure 4A). The bright-field illumination channel was used to determine hypha boundaries and to calculate their surface area. Using the red fluorescence channel of the microscopy images, EcN could then be observed, and the total area of bacteria was determined. Based on these area measurements, we defined different parameters which are indicated in Table S3. The most adequate parameter to consider is the percent of hyphae covered with bacteria, which is the calculation of the overlap area between hyphae and bacteria divided by the total area of hyphae. This value represents a quantitative measurement of EcN attachment to *C. albicans* hyphae.

Next, this tool was utilized to quantify the attachment of different EcN strains to hyphae. To this end, three different images at random locations on the microscope slide were used for each strain. This experiment was performed three times on different days, and the percent of hyphae covered with bacteria was calculated for each image, which resulted in the analysis of nine images per strain. These nine values as well as their average are plotted in Figure 4B. In accordance with previous visual observation, both EcN/chi and EcN/SP1 showed approximately 2.5% of hyphae covered with bacteria, whereas the control strain, EcN/act, EcN/dec, and EcN/SP2 displayed less than 0.5% of hyphae covered with bacteria (*p* < 0.001 for EcN/chi and EcN/SP1). On the other hand, EcN/act, EcN/dec, and EcN/SP2 did not show any significant difference when compared to EcN/control. It is also interesting to note that no significant differences could be observed between the values obtained for EcN/SP1 and EcN/chi, indicating that both strains have similar affinities for hyphae. Additionally, a similar experiment was conducted with bacteria and hyphae coincubated for only 4 h (Figure S6). In this setup, only EcN/SP1 showed 2% of hyphae covered by bacteria, whereas EcN/chi did not display binding.

The lack of adhesion between hyphae and EcN/dec can be explained by the very low production of recombinant protein shown by the Western blot experiment and the subsequent absence of a quantifiable protein display. Similarly, for EcN/act, the absence of adhesion tends to confirm the hypothesis drawn from the antibody labeling experiment that the recombinant protein was not displayed on the cell surface even though high production could be demonstrated by Western blot. Interestingly, EcN/SP2, which produced high amounts of fusion protein and showed a high degree of surface display, did not bind to *C. albicans* hyphae, thus providing a negative control protein displayed via the AIDA system. The only strains that could achieve hyphae attachment were EcN/chi and EcN/SP1, the latter with fast attachment after 4 h. Attachment of the chitinase 92-displaying strain was expected as the binding domain of this strain possesses affinity for chitin, one of the major components of the *C. albicans* cell wall. SP1, on the other hand, is a randomly chosen polypeptide of the same size as the chitinase 92 binding domain, and its binding affinity for *C. albicans* was not anticipated. However, as already stated, carbohydrate binding of proteins is mediated by hydrophobic interactions involving aromatic amino acids. It was previously shown that the aromatic residues tryptophan, tyrosine, and, to a lesser extent, histidine are over-represented in carbohydrate binding domains, and the implication of these residues for sugar binding by invoking electronic and electrostatic considerations was discussed.^58^ Chitinase 92 itself was shown to rely on aromatic amino acids for binding to chitin as the replacement of key tryptophan residues by alanine drastically reduced the affinity to chitin.^70^ Moreover, the topology of the carbohydrate binding domain plays a very important role, with the presence of several β-sheets in a planar architecture being a characteristic of crystalline chitin binding of carbohydrate binding modules of type A.^71^ In the N-terminal part of the SP1-AIDA fusion, one tryptophan and three tyrosine residues are present, and protein modeling suggests that this N-terminal part is readily available for binding interactions. Additionally, protein structure prediction indicated that the binding domain of SP1-AIDA may contain up to seven β-sheets with four of them arranged as a planar surface, resembling type A carbohydrate binding modules (Figure S5). In SP2, all aromatic residues were exchanged for aliphatic amino acids, and other mutations were performed, which completely abolished the presence of β-sheets in the binding domain and resulted in the complete loss of hyphae adhesion. This led to the hypothesis that the presence of aromatic amino acids and the possibility to form β-sheets in a planar architecture are responsible for the high affinity of SP1-AIDA toward *C. albicans* hyphae.

### Binding to *C. albicans* in Yeast Form

**Figure 5:**
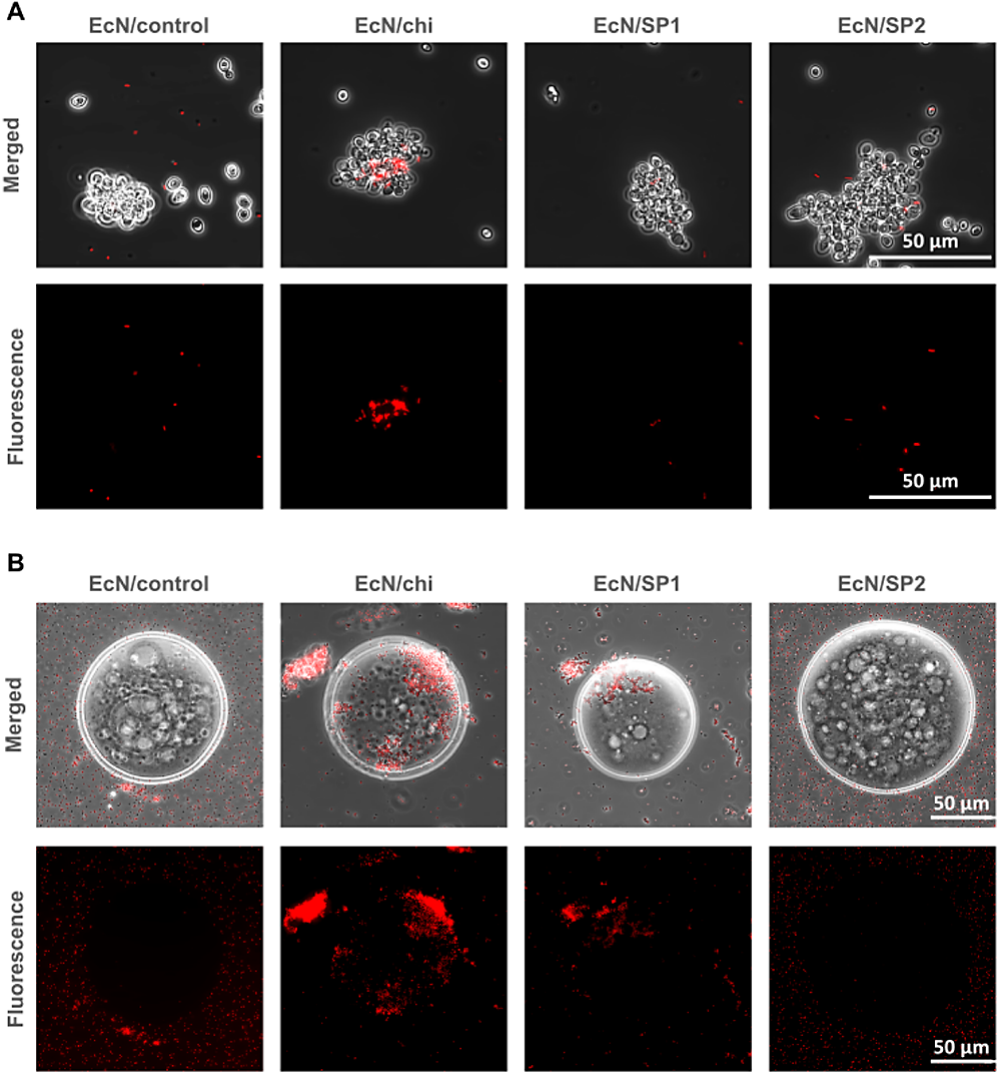
(A) Representative microscope images after the incubation of the indicated strains with *C. albicans* in the yeast form. Images presented are a merger of the bright-field and red fluorescence channel (top) or only the red fluorescence channel (bottom). (B) Representative microscope images after incubation of the indicated strains with chitin beads. Images presented are a merger of the bright-field and red fluorescence channel (top) or only the red fluorescence channel (bottom).

We were interested in testing if the strains that are able to attach to *C. albicans* hyphae can also bind to its yeast form. The cell wall composition of *C. albicans* is modified during the yeast-to-hyphae transition with notably the proportion of chitin increasing from 1–2 % to 10–20%.^43^ Unfortunately, the smaller size of *C. albicans* in the yeast form does not allow a simple separation of yeast from EcN as was the case for hyphae. After the mixing of EcN with *C. albicans* and subsequent incubation, a sample of this mixture was therefore observed under the microscope without separation. This setup implied to carefully optimize the amount of each partner in the mix. To enable accurate interpretation, it was crucial to ensure a sufficient number of bacteria encounter yeast cells, while also avoiding an excess that could make it impossible to distinguish between binders and nonbinders. A representative microscopy image of the combination between *C. albicans* yeast cells and EcN/control, EcN/chi, EcN/SP1, and EcN/SP2 is depicted in Figure 5A. From these images, the characteristically shaped yeast cells are easily distinguishable from red fluorescent EcN cells. Besides free-floating cells, some *C. albicans* cells also aggregate into clumps, a phenomenon which is independent of mixing with bacterial cells as yeast cells alone do aggregate after some hours in PBS at 37 °C. Clear colocalization of bacteria and *C. albicans* yeast cells could only be observed with EcN/chi. Interestingly, this binding seems to occur inside the yeast aggregates, and no attachment could be observed between free-floating yeast and EcN/chi. For the other EcN strains, no colocalization of bacteria with yeast aggregates or free-floating *C. albicans* cells was observed. From this result, it can be inferred that although both EcN/chi and EcN/SP1 attach to *C. albicans* hyphae, they may recognize different structures of the cell wall. EcN/chi has an affinity for both the yeast and the hyphae, whereas EcN/SP1 shows an affinity for only the hyphae form.

Given the difficulties to study binding interactions between yeast cells and our modified EcN strains, utilization of alternative techniques like flow cytometry can be envisioned; nevertheless, their likelihood of success in our setup is minimal. While this method has been used for the study of *S. cerevisiae*–*E. coli* binding,^72^ its implementation was shown to be possible only when high-affinity molecules were expressed on the surface of both organisms, namely, nanobodies and their cognate antigens. Moreover, yeast aggregation often necessitates vortexing steps in order to obtain clear flow cytometry results, and these vortexing steps may jeopardize the interactions between our *E. coli* Nissle and *C. albicans* cells.

### Binding to Abiotic Surfaces

Because the last experiment hinted that the two bacterial strains EcN/chi and EcN/SP1 seem to recognize different structures on the cell wall of *C. albicans*, we planned to investigate their affinity more precisely by incubating them with different surfaces, containing carbohydrates or not. To keep a comparable experimental setup, we used an abiotic surface in the form of nonsoluble small-sized particles of dimensions below 300 μm. Adhesion to chitin beads, cellulose, and polyethylene terephthalate (PET) was assessed to test whether EcN/chi and EcN/SP1 can recognize, respectively, chitin from the cell wall of *C. albicans*, polymers made of carbohydrates absent in *C. albicans*, and polymers without carbohydrates. After incubation of the different bacterial strains with these abiotic surfaces, the mixture was not passed through a strainer because it caused clogging of the nylon mesh. Similar assays with a strainer possessing a PET mesh resulted in the same issues. As a consequence, 6 μL of the bacteria/abiotic surface mixture was directly observed under the microscope without separation.

Chitin beads are spherical particles of β-1,4-linked *N*-acetylglucosamine ranging in size between 50 and 100 μm. They are routinely used in affinity chromatography to capture recombinant proteins possessing a chitin binding domain from chitinase A1.^73^ Representative microscope images of the bacteria/chitin bead mixture after 18 h of incubation at 37 °C are shown in Figure 5B. A clear attachment of EcN/chi to the surface of chitin beads could be observed, whereas EcN/control and EcN/SP2 were only marginally colocalizing with the chitin beads. EcN/SP1 also displayed some attachment to the beads, however, to a lesser degree. It is important to note that for this assay, a higher titer of bacterial cells was used than for the previous experiments. Preliminary tests indicated that, even though attachment of EcN/chi was visible with a low number of bacteria, the effect was more noticeable when this number was increased. Interestingly, EcN/chi and EcN/SP1 but not the two other strains showed some degree of self-aggregation. From this experimental setup, it cannot be determined if self-binding occurred because the membrane-displayed proteins tend to self-aggregate or if these proteins bind to natural components of EcN. The fact that EcN/SP2 did not exhibit this phenotype indicates that AIDA display itself is not sufficient to promote cell aggregation. Nevertheless, this experiment strengthens the hypothesis that EcN/chi recognizes and binds chitin. On the other hand, EcN/SP1 seems to have a lower affinity for chitin than EcN/chi, and its strong binding to fungal cell walls might involve other cell wall components. Interestingly, binding of modified *E. coli* BL21 to chitin beads was already performed before.^74^ In their setup, the authors of this study utilized the lpp-ompA display system to expose the carbohydrate binding domain of chitinase A1, a protein from *Bacillus circulans* possessing a carbohydrate binding module of family 12 (CBM12) and which is distantly related to proteins having a carbohydrate binding module of family 5 (CBM5), to which belongs chi92.^71^

After demonstrating the affinity of the engineered bacteria for fungal hyphae, we also investigated the specificity of this binding capacity. Low specificity, for example, binding to any hydrophobic surface, could significantly impede the performance of a therapeutic microbe. Therefore, we tested our hyphae binding strains for affinity to the abundant plant cell wall component cellulose and the commonly used polyester material polyethylene terephthalate (PET). Representing one of the major fiber components in plant-based foods, cellulose is both bound and degraded by certain bacteria in the human intestinal tract.^75,76^ To investigate the attachment of modified EcN strains to cellulose, a similar experiment to that for chitin was performed. As depicted in Figure S7, some bacteria were localized on the surface of the cellulose particles, but no obvious differences between the strains could be observed.

PET is one of the most widely used petroleum-based polyesters and is commercialized as a powder of different sizes. This synthetic polymer has already been detected in human feces in the form of microparticles due to their extensive use in the food packaging industry and their very slow degradation.^77,78^ We performed a binding assay with our modified EcN strains and found no obvious difference in PET binding capacity between the control strain and our hyphae binding strains (Figure S8). While it is impossible to exclude the possibility that the engineered strains exhibit enhanced binding to materials other than components present in the fungal cell wall, these two experiments illustrate that engineered EcN/chi and EcN/SP1 do not generally exhibit enhanced adherence to surfaces, irrespective of their composition. Therefore, they should not be inferior to the wild-type EcN strain concerning their tendency to involve in unspecific surface attachment, despite their enhanced hyphae binding capacity.

Besides abiotic surfaces, LBPs in the digestive tract will encounter many different living cells, in particular, host cells and members of our microbiome. Given the very high inter-individual diversity of the microbiome and the strikingly different cell wall that these members of the microbiome possess, it would be unpracticable to test their adhesion to our EcN-based LBPs.^79^*S. cerevisiae*, which possess a very similar cell wall compared to the yeast form of *C. albicans*, would probably show a comparable adhesion, but this organism is often absent from the gut mycobiome, and the mycobiome itself only represents about 0.1% of the total microbiome.^80^ Moreover, even if off-target binding will likely occur, the implementation of target-specific production of active compounds thanks to the utilization of quorum sensing induced promoter, for example, will help to ensure selectivity of the final LBP.

## Conclusion

In this work, we engineered the probiotic strain *E. coli* Nissle to attach to the virulence-associated hyphal form of the fungal pathogen *C. albicans*. The observed adhesive property was implemented by the surface display of carbohydrate binding proteins via the AIDA-display system. During the screening of different passenger proteins, we found that displaying the carbohydrate binding domain of the chitinase 92 protein and the synthetic peptide SP1 conferred the ability to adhere to *C. albicans* hyphae without enhancing binding affinity to the yeast form or to selected control polymers. With the help of a newly developed algorithm-based image analysis tool, we were able to quantify the attachment of bacteria to hyphae, a methodology that could be very useful for related projects. We believe that engineering surface binding modules can, in principle, dramatically increase the potential of LBPs when combined with the delivery of effector molecules. Such a “sticky” therapeutic microbe should outperform a nonadhering strain by reaching higher local effector concentrations. In the particular case of *C. albicans* hyphae, the delivery of antivirulence factors or antifungal compounds could directly target the invasive filamentous form of *Candida*. This approach could reduce the emergence of resistance and minimize side effects on the host, as well as on members of its microbiome. Therefore, this study opens up fascinating perspectives for exploring the full therapeutic potential of bioengineered probiotics.

## Supporting Information Available

Supporting tables listing genetic parts, sequencing primers, and JIPipe parameters; supporting figures providing additional data about the colibactin gene cluster deletion, immunolabelling of non-FLAG-tagged strains, immunolabelling of engineered *E. coli* BL21 strains, structure predictions of fusion proteins, adhesion to *C. albicans* after 4 h, and adhesion to abiotic surfaces (PDF)
Supporting file containing all plasmid sequences in GenBank format (ZIP)

## References

[ref1] Bongomin F.; Gago S.; Oladele R. O.; Denning D. W. Global and multi-national prevalence of fungal diseases-estimate precision. J. Fungi 2017, 3, 57 10.3390/jof3040057. PMC5753159 29371573

[ref2] Nnadi N. E.; Carter D. A. Climate change and the emergence of fungal pathogens. PLoS Pathog. 2021, 17, e1009503 10.1371/journal.ppat.1009503. 33914854 PMC8084208

[ref3] Casadevall A.; Kontoyiannis D. P.; Robert V. On the emergence of *Candida auris*: Climate change, azoles, swamps, and birds. mBio 2019, 10, 10–1128. 10.1128/mBio.01397-19. PMC6650554 31337723

[ref4] World Health Organization. Who fungal priority pathogens list to guide research, development and public health action, World Health Organization, 2022. https://www.who.int/publications/i/item/9789240060241.

[ref5] Lopes J. P.; Lionakis M. S. Pathogenesis and virulence of *Candida albicans*. Virulence 2022, 13, 89–121. 10.1080/21505594.2021.2019950. 34964702 PMC9728475

[ref6] Kumamoto C. A.; Gresnigt M. S.; Hube B. The gut, the bad and the harmless: *Candida albicans* as a commensal and opportunistic pathogen in the intestine. Curr. Opin. Microbiol. 2020, 56, 7–15. 10.1016/j.mib.2020.05.006. 32604030 PMC7744392

[ref7] Denning D. W. Antifungal drug resistance: An update. Eur. J. Hosp. Pharm. 2022, 29, 109–112. 10.1136/ejhpharm-2020-002604. 35190454 PMC8899664

[ref8] Cubillos-Ruiz A.; Guo T.; Sokolovska A.; Miller P. F.; Collins J. J.; Lu T. K.; Lora J. M. Engineering living therapeutics with synthetic biology. Nat. Rev. Drug Discovery 2021, 20, 941–960. 10.1038/s41573-021-00285-3. 34616030

[ref9] Rottinghaus A. G.; Amrofell M. B.; Moon T. S. Biosensing in smart engineered probiotics. Biotechnol. J. 2020, 15, 1900319 10.1002/biot.201900319. PMC7305048 31860168

[ref10] Zhou Z.; Chen X.; Sheng H.; Shen X.; Sun X.; Yan Y.; Wang J.; Yuan Q. Engineering probiotics as living diagnostics and therapeutics for improving human health. Microb. Cell Fact. 2020, 19, 56 10.1186/s12934-020-01318-z. 32131831 PMC7055047

[ref11] Landry B. P.; Tabor J. J. Engineering diagnostic and therapeutic gut bacteria. Microbiol. Spectr. 2017, 5, 331–361. 10.1128/microbiolspec.BAD-0020-2017. PMC11687543 29052539

[ref12] Harimoto T.; Hahn J.; Chen Y.-Y.; Im J.; Zhang J.; Hou N.; Li F.; Coker C.; Gray K.; Harr N.; Chowdhury S.; Pu K.; Nimura C.; Arpaia N.; Leong K. W.; Danino T. A programmable encapsulation system improves delivery of therapeutic bacteria in mice. Nat. Biotechnol. 2022, 40, 1259–1269. 10.1038/s41587-022-01244-y. 35301496 PMC9371971

[ref13] Hwang I. Y.; Koh E.; Wong A.; March J. C.; Bentley W. E.; Lee Y. S.; Chang M. W. Engineered probiotic *Escherichia coli* can eliminate and prevent *Pseudomonas aeruginosa* gut infection in animal models. Nat. Commun. 2017, 11, 15028 10.1038/ncomms15028. PMC5394271 28398304

[ref14] Palmer J. D.; Piattelli E.; McCormick B. A.; Silby M. W.; Brigham C. J.; Bucci V. Engineered probiotic for the inhibition of *Salmonella* via tetrathionate-induced production of microcin h47. ACS Infect. Dis. 2018, 4, 39–45. 10.1021/acsinfecdis.7b00114. 28918634 PMC5766358

[ref15] Tscherner M.; Giessen T. W.; Markey L.; Kumamoto C. A.; Silver P. A. A synthetic system that senses *Candida albicans* and inhibits virulence factors. ACS Synth. Biol. 2019, 8, 434–444. 10.1021/acssynbio.8b00457. 30608638

[ref16] Brooks S. M.; Alper H. S. Applications, challenges, and needs for employing synthetic biology beyond the lab. Nat. Commun. 2021, 12, 1390 10.1038/s41467-021-21740-0. 33654085 PMC7925609

[ref17] McNerney M. P.; Doiron K. E.; Ng T. L.; Chang T. Z.; Silver P. A. Theranostic cells: Emerging clinical applications of synthetic biology. Nat. Rev. Genet. 2021, 22, 730–746. 10.1038/s41576-021-00383-3. 34234299 PMC8261392

[ref18] Salazar S. B.; Simoes R. S.; Pedro N. A.; Pinheiro M. J.; Carvalho M. F. N. N.; Mira N. P. An overview on conventional and non-conventional therapeutic approaches for the treatment of candidiasis and underlying resistance mechanisms in clinical strains. J. Fungi 2020, 6, 23 10.3390/jof6010023. PMC7151124 32050673

[ref19] Gintjee T. J.; Donnelley M. A.; Thompson G. R. Aspiring antifungals: Review of current antifungal pipeline developments. J. Fungi 2020, 6, 28 10.3390/jof6010028. PMC7151215 32106450

[ref20] Cravens A.; Payne J.; Smolke C. D. Synthetic biology strategies for microbial biosynthesis of plant natural products. Nat. Commun. 2019, 10, 2142 10.1038/s41467-019-09848-w. 31086174 PMC6513858

[ref21] Li L.; Maclntyre L. W.; Brady S. F. Refactoring biosynthetic gene clusters for heterologous production of microbial natural products. Curr. Opin. Biotechnol. 2021, 69, 145–152. 10.1016/j.copbio.2020.12.011. 33476936 PMC8238852

[ref22] Chen H.; Lei P.; Ji H.; Yang Q.; Peng B.; Ma J.; Fang Y.; Qu L.; Li H.; Wu W.; Jin L.; Sun D. Advances in *Escherichia coli* nissle 1917 as a customizable drug delivery system for disease treatment and diagnosis strategies. Mater. Today Bio 2023, 18, 100543 10.1016/j.mtbio.2023.100543. PMC9840185 36647536

[ref23] Fietto J. L.; Araújo R. S.; Valadão F. N.; Fietto L. G.; Brandão R. L.; Neves M. J.; Gomes F. C.; Nicoli J. R.; Castro I. M. Molecular and physiological comparisons between Saccharomyces cerevisiae and Saccharomyces boulardii. Can. J. Microbiol. 2004, 50, 615–621. 10.1139/w04-050. 15467787

[ref24] Zhao L.; Yin G.; Zhang Y.; Duan C.; Wang Y.; Kang Z. A comparative study on the genomes, transcriptomes, and metabolic properties of *Escherichia coli* strains nissle 1917, bl21(de3), and mg1655. Eng. Microbiol. 2022, 2, 100012 10.1016/j.engmic.2022.100012. PMC11610980 39628614

[ref25] Olvera-Rosales L. B.; Cruz-Guerrero A. E.; Ramírez-Moreno E.; Quintero-Lira A.; Contreras-López E.; Jaimez-Ordaz J.; Castañeda-Ovando A.; Añorve-Morga J.; Calderón-Ramos Z. G.; Arias-Rico J. Impact of the Gut Microbiota Balance on the Health–Disease Relationship: The Importance of Consuming Probiotics and Prebiotics. Foods 2021, 10, 1261 10.3390/foods10061261. 34199351 PMC8230287

[ref26] Park S. J.; Han K. H.; Park J. Y.; Choi S. J.; Lee K. H. Influence of bacterial presence on biofilm formation of *Candida albicans*. Yonsei Med. J. 2014, 55, 449–458. 10.3349/ymj.2014.55.2.449. 24532517 PMC3936627

[ref27] Piñero-Lambea C.; Bodelón G.; Fernández-Periáñez R.; Cuesta A. M.; Álvarez-Vallina L.; Fernández L. Á. Programming controlled adhesion of *E. coli* to target surfaces, cells, and tumors with synthetic adhesins. ACS Synth. Biol. 2015, 4, 463–473. 10.1021/sb500252a. 25045780 PMC4410913

[ref28] Ho C. L.; Tan H. Q.; Chua K. J.; Kang A.; Lim K. H.; Ling K. L.; Yew W. S.; Lee Y. S.; Thiery J. P.; Chang M. W. Engineered commensal microbes for diet-mediated colorectal-cancer chemoprevention. Nat. Biomed. Eng. 2018, 2, 27–37. 10.1038/s41551-017-0181-y. 31015663

[ref29] Zhang Z.; Meng L.; Ni C.; Yao L.; Zhang F.; Jin Y.; Mu X.; Zhu S.; Lu X.; Liu S.; Yu C.; Wang C.; Zheng P.; Wu J.; Kang L.; Zhang H. M.; Ouyang Q. Engineering *Escherichia coli* to bind to cyanobacteria. J. Biosci. Bioeng. 2017, 123, 347–352. 10.1016/j.jbiosc.2016.09.010. 27773604

[ref30] Nicchi S.; Giuliani M.; Giusti F.; Pancotto L.; Maione D.; Delany I.; Galeotti C. L.; Brettoni C. Decorating the surface of *Escherichia coli* with bacterial lipoproteins: A comparative analysis of different display systems. Microb. Cell Fact. 2021, 20, 33 10.1186/s12934-021-01528-z. 33531008 PMC7853708

[ref31] van Bloois E.; Winter R. T.; Kolmar H.; Fraaije M. W. Decorating microbes: Surface display of proteins on *Escherichia coli*. Trends Biotechnol. 2011, 29, 79–86. 10.1016/j.tibtech.2010.11.003. 21146237

[ref32] Kim D.; Ku S. Bacillus cellulase molecular cloning, expression, and surface display on the outer membrane of *Escherichia coli*. Molecules 2018, 23, 503 10.3390/molecules23020503. 29495265 PMC6017809

[ref33] Gao F.; Ding H.; Feng Z.; Liu D.; Zhao Y. Functional display of triphenylmethane reductase for dye removal on the surface of *Escherichia coli* using n-terminal domain of ice nucleation protein. Bioresour. Technol. 2014, 169, 181–187. 10.1016/j.biortech.2014.06.093. 25058292

[ref34] Gustavsson M.; Bäcklund E.; Larsson G. Optimisation of surface expression using the aida autotransporter. Microb. Cell Fact. 2011, 10, 72 10.1186/1475-2859-10-72. 21917130 PMC3192670

[ref35] Jarmander J.; Gustavsson M.; Do T.-H.; Samuelson P.; Larsson G. A dual tag system for facilitated detection of surface expressed proteins in *Escherichia coli*. Microb. Cell Fact. 2012, 11, 118 10.1186/1475-2859-11-118. 22943700 PMC3511212

[ref36] Chmielewski M.; Kuehle J.; Chrobok D.; Riet N.; Hallek M.; Abken H. Fimh-based display of functional eukaryotic proteins on bacteria surfaces. Sci. Rep. 2019, 9, 8410 10.1038/s41598-019-44883-z. 31182802 PMC6557881

[ref37] Sonnenborn U.; Schulze J. The non-pathogenic *Escherichia coli* strain nissle 1917 – features of a versatile probiotic. Microb. Ecol. Health Dis. 2009, 21, 122–158. 10.3109/08910600903444267.

[ref38] Kan A.; Gelfat I.; Emani S.; Praveschotinunt P.; Joshi N. S. Plasmid vectors for in vivo selection-free use with the probiotic *E. coli* nissle 1917. ACS Synth. Biol. 2021, 10, 94–106. 10.1021/acssynbio.0c00466. 33301298 PMC7813132

[ref39] Vaaben T. H.; Vazquez-Uribe R.; Sommer M. O. A. Characterization of eight bacterial biosensors for microbial diagnostic and therapeutic applications. ACS Synth. Biol. 2022, 11, 4184–4192. 10.1021/acssynbio.2c00491. 36449712 PMC9764412

[ref40] Netea M. G.; Brown G. D.; Kullberg B. J.; Gow N. A. An integrated model of the recognition of *Candida albicans* by the innate immune system. Nat. Rev. Microbiol. 2008, 6, 67–78. 10.1038/nrmicro1815. 18079743

[ref41] Goyal S.; Castrillon-Betancur J. C.; Klaile E.; Slevogt H. The interaction of human pathogenic fungi with c-type lectin receptors. Front. Immunol. 2018, 9, 1261 10.3389/fimmu.2018.01261. 29915598 PMC5994417

[ref42] Cottier F.; Hall R. A. Face/off: The interchangeable side of *Candida albicans*. Front. Cell. Infect. Microbiol. 2019, 9, 471 10.3389/fcimb.2019.00471. 32047726 PMC6997470

[ref43] Garcia-Rubio R.; de Oliveira H. C.; Rivera J.; Trevijano-Contador N. The fungal cell wall: *Candida*, *Cryptococcus*, and *Aspergillus* species. Front. Microbiol. 2020, 10, 2993 10.3389/fmicb.2019.02993. 31993032 PMC6962315

[ref44] Gow N. A. R.; Latge J. P.; Munro C. A. The fungal cell wall: Structure, biosynthesis, and function. Microbiol. Spectr. 2017, 5, 10–1128. 10.1128/microbiolspec.FUNK-0035-2016. PMC11687499 28513415

[ref45] Peters B. M.; Ovchinnikova E. S.; Krom B. P.; Schlecht L. M.; Zhou H.; Hoyer L. L.; Busscher H. J.; van der Mei H. C.; Jabra-Rizk M. A.; Shirtliff M. E. *Staphylococcus aureus* adherence to *Candida albicans* hyphae is mediated by the hyphal adhesin als3p. Microbiology 2012, 158, 2975–2986. 10.1099/mic.0.062109-0. 22918893 PMC4083660

[ref46] Brand A.; Barnes J. D.; Mackenzie K. S.; Odds F. C.; Gow N. A. R. Cell wall glycans and soluble factors determine the interactions between the hyphae of *Candida albicans* and *Pseudomonas aeruginosa*. FEMS Microbiol. Lett. 2008, 287, 48–55. 10.1111/j.1574-6968.2008.01301.x. 18680523 PMC2613227

[ref47] Ovchinnikova E. S.; Krom B. P.; Harapanahalli A. K.; Busscher H. J.; van der Mei H. C. Surface thermodynamic and adhesion force evaluation of the role of chitin-binding protein in the physical interaction between *Pseudomonas aeruginosa* and *Candida albicans*. Langmuir 2013, 29, 4823–4829. 10.1021/la400554g. 23509956

[ref48] Reisch C. R.; Prather K. L. J. The no-scar (scarless cas9 assisted recombineering) system for genome editing in *Escherichia coli*. Sci. Rep. 2015, 5, 15096 10.1038/srep15096. 26463009 PMC4604488

[ref49] Schneider C. A.; Rasband W. S.; Eliceiri K. W. Nih image to imagej: 25 years of image analysis. Nat. Methods 2012, 9, 671–675. 10.1038/nmeth.2089. 22930834 PMC5554542

[ref50] Gerst R.; Cseresnyés Z.; Figge M. T. Jipipe: Visual batch processing for imagej. Nat. Methods 2023, 20, 168–169. 10.1038/s41592-022-01744-4. 36627450

[ref51] Mirdita M.; Schütze K.; Moriwaki Y.; Heo L.; Ovchinnikov S.; Steinegger M. Colabfold: Making protein folding accessible to all. Nat. Methods 2022, 19, 679–682. 10.1038/s41592-022-01488-1. 35637307 PMC9184281

[ref52] Sehnal D.; Bittrich S.; Deshpande M.; Svobodová R.; Berka K.; Bazgier V.; Velankar S.; Burley S. K.; Koča J.; Rose A. S. Mol* viewer: Modern web app for 3d visualization and analysis of large biomolecular structures. Nucleic Acids Res. 2021, 49, W431–W437. 10.1093/nar/gkab314. 33956157 PMC8262734

[ref53] Anderson J. C., Promoters/catalog/anderson, 2006. http://parts.igem.org/Promoters/Catalog/Anderson.

[ref54] Wu M. L.; Tsai C. Y.; Chen T. H. Cell surface display of chi92 on *Escherichia coli* using ice nucleation protein for improved catalytic and antifungal activity. FEMS Microbiol. Lett. 2006, 256, 119–125. 10.1111/j.1574-6968.2006.00115.x. 16487328

[ref55] Chiba H.; Inokoshi J.; Nakashima H.; Omura S.; Tanaka H. Actinohivin, a novel anti-human immunodeficiency virus protein from an actinomycete, inhibits viral entry to cells by binding high-mannose type sugar chains of gp120. Biochem. Biophys. Res. Commun. 2004, 316, 203–210. 10.1016/j.bbrc.2004.02.036. 15003531

[ref56] McGreal E. P.; Rosas M.; Brown G. D.; Zamze S.; Wong S. Y.; Gordon S.; Martinez-Pomares L.; Taylor P. R. The carbohydrate-recognition domain of dectin-2 is a c-type lectin with specificity for high mannose. Glycobiology 2006, 16, 422–430. 10.1093/glycob/cwj077. 16423983

[ref57] Duvaud S.; Gabella C.; Lisacek F.; Stockinger H.; Ioannidis V.; Durinx C. Expasy, the swiss bioinformatics resource portal, as designed by its users. Nucleic Acids Res. 2021, 49, W216–W227. 10.1093/nar/gkab225. 33849055 PMC8265094

[ref58] Hudson K. L.; Bartlett G. J.; Diehl R. C.; Agirre J.; Gallagher T.; Kiessling L. L.; Woolfson D. N. Carbohydrate-aromatic interactions in proteins. J. Am. Chem. Soc. 2015, 137, 15152–15160. 10.1021/jacs.5b08424. 26561965 PMC4676033

[ref59] Burdette L. A.; Leach S. A.; Wong H. T.; Tullman-Ercek D. Developing gram-negative bacteria for the secretion of heterologous proteins. Microb. Cell Fact. 2018, 17, 196 10.1186/s12934-018-1041-5. 30572895 PMC6302416

[ref60] Suhr M.; Benz I.; Schmidt M. A. Processing of the aida-i precursor: Removal of aidac and evidence for the outer membrane anchoring as a β-barrel structure. Mol. Microbiol. 1996, 22, 31–42. 10.1111/j.1365-2958.1996.tb02653.x. 8899706

[ref61] Charbonneau M. E.; Berthiaume F.; Mourez M. Proteolytic processing is not essential for multiple functions of the *Escherichia coli* autotransporter adhesin involved in diffuse adherence (AIDA-I. J. Bacteriol. 2006, 188, 8504–8512. 10.1128/JB.00864-06. 17041044 PMC1698232

[ref62] Charbonneau M.; Janvore J.; Mourez M. Autoprocessing of the *Escherichia coli* AIDA-I autotransporter: A new mechanism involving acidic residues in the junction region. J. Biol. Chem. 2009, 284, 17340–17351. 10.1074/jbc.M109.010108. 19398552 PMC2719369

[ref63] Nougayrède J.-P.; Chagneau C. V.; Motta J.-P.; Bossuet-Greif N.; Belloy M.; Taieb F.; Gratadoux J.-J.; Thomas M.; Langella P.; Oswald E. A toxic friend: Genotoxic and mutagenic activity of the probiotic strain *Escherichia coli* nissle 1917. mSphere 2021, 6, 10–1128. 10.1128/mSphere.00624-21. PMC8386472 34378987

[ref64] Lobstein J.; Emrich C. A.; Jeans C.; Faulkner M.; Riggs P.; Berkmen M. Shuffle, a novel *Escherichia coli* protein expression strain capable of correctly folding disulfide bonded proteins in its cytoplasm. Microb. Cell Fact. 2012, 11, 56 10.1186/1475-2859-11-56. 22569138 PMC3526497

[ref65] Feinberg H.; Jégouzo S. A. F.; Rex M. J.; Drickamer K.; Weis W. I.; Taylor M. E. Mechanism of pathogen recognition by human dectin-2. J. Biol. Chem. 2017, 292, 13402–13414. 10.1074/jbc.M117.799080. 28652405 PMC5555199

[ref66] Sarnelli G.; Del Re A.; Pesce M.; Lu J.; Esposito G.; Sanseverino W.; Corpetti C.; Basili Franzin S.; Seguella L.; Palenca I.; Rurgo S.; De Palma F. D. E.; Zilli A.; Esposito G. Oral immunization with *Escherichia coli* nissle 1917 expressing sars-cov-2 spike protein induces mucosal and systemic antibody responses in mice. Biomolecules 2023, 13, 569 10.3390/biom13030569. 36979504 PMC10046078

[ref67] Ninyio N.; Schmitt K.; Sergon G.; Nilsson C.; Andersson S.; Scherbak N. Stable expression of hiv-1 mper extended epitope on the surface of the recombinant probiotic bacteria *Escherichia coli* nissle 1917 using crispr/cas9. Microb. Cell Fact. 2024, 23, 75 10.1186/s12934-024-02347-8. 38448924 PMC10918952

[ref68] Buddenborg C.; Daudel D.; Liebrecht S.; Greune L.; Humberg V.; Schmidt M. A. Development of a tripartite vector system for live oral immunization using a gram-negative probiotic carrier. Int. J. Med. Microbiol. 2008, 298, 105–114. 10.1016/j.ijmm.2007.08.008. 17936683

[ref69] Jumper J.; Evans R.; Pritzel A.; Green T.; Figurnov M.; Ronneberger O.; Tunyasuvunakool K.; Bates R.; Žídek A.; Potapenko A.; Bridgland A.; Meyer C.; Kohl S. A. A.; Ballard A. J.; Cowie A.; Romera-Paredes B.; Nikolov S.; Jain R.; Adler J.; Back T.; Petersen S.; Reiman D.; Clancy E.; Zielinski M.; Steinegger M.; Pacholska M.; Berghammer T.; Bodenstein S.; Silver D.; Vinyals O.; Senior A. W.; Kavukcuoglu K.; Kohli P.; Hassabis D. Highly accurate protein structure prediction with alphafold. Nature 2021, 596, 583–589. 10.1038/s41586-021-03819-2. 34265844 PMC8371605

[ref70] Chang M. C.; Lai P. L.; Wu M. L. Biochemical characterization and site-directed mutational analysis of the double chitin-binding domain from chitinase 92 of *Aeromonas hydrophila* jp101. FEMS Microbiol. Lett. 2004, 232, 61–66. 10.1016/S0378-1097(04)00014-X. 15019735

[ref71] Boraston A. B.; Bolam D. N.; Gilbert H. J.; Davies G. J. Carbohydrate-binding modules: Fine-tuning polysaccharide recognition. Biochem. J. 2004, 382, 769–781. 10.1042/BJ20040892. 15214846 PMC1133952

[ref72] Lei M.; Trivedi V. D.; Nair N. U.; Lee K.; Van Deventer J. A. Flow cytometric evaluation of yeast-bacterial cell-cell interactions. Biotechnol. Bioeng. 2023, 120, 399–408. 10.1002/bit.28253. 36259110 PMC10072783

[ref73] Mitchell S. F.; Lorsch J. R. Protein affinity purification using intein/chitin binding protein tags. Methods Enzymol. 2015, 559, 111–125. 10.1016/bs.mie.2014.11.002. 26096506

[ref74] Wang J.-Y.; Chao Y.-P. Immobilization of cells with surface-displayed chitin-binding domain. Appl. Environ. Microbiol. 2006, 72, 927–931. 10.1128/AEM.72.1.927-931.2006. 16391137 PMC1352180

[ref75] Chassard C.; Delmas E.; Robert C.; Bernalier-Donadille A. The cellulose-degrading microbial community of the human gut varies according to the presence or absence of methanogens. FEMS Microbiol. Ecol. 2010, 74, 205–213. 10.1111/j.1574-6941.2010.00941.x. 20662929

[ref76] Liu Z.; Liu H.; Vera A. M.; Bernardi R. C.; Tinnefeld P.; Nash M. A. High force catch bond mechanism of bacterial adhesion in the human gut. Nat. Commun. 2020, 11, 4321 10.1038/s41467-020-18063-x. 32859904 PMC7456326

[ref77] Tamargo A.; Molinero N.; Reinosa J. J.; Alcolea-Rodriguez V.; Portela R.; Bañares M. A.; Fernández J. F.; Moreno-Arribas M. V. Pet microplastics affect human gut microbiota communities during simulated gastrointestinal digestion, first evidence of plausible polymer biodegradation during human digestion. Sci. Rep. 2022, 12, 528 10.1038/s41598-021-04489-w. 35017590 PMC8752627

[ref78] Schwabl P.; Köppel S.; Königshofer P.; Bucsics T.; Trauner M.; Reiberger T.; Liebmann B. Detection of various microplastics in human stool: A prospective case series. Ann. Int. Med. 2019, 171, 453–457. 10.7326/M19-0618. 31476765

[ref79] Huse S. M.; Ye Y.; Zhou Y.; Fodor A. A. A core human microbiome as viewed through 16s rrna sequence clusters. PLoS One 2012, 7, e34242 10.1371/journal.pone.0034242. 22719824 PMC3374614

[ref80] Belvoncikova P.; Splichalova P.; Videnska P.; Gardlik R. The human mycobiome: Colonization, composition and the role in health and disease. J. Fungi 2022, 8, 1046 10.3390/jof8101046. PMC9605233 36294611

