# Supplementary material for: Engineering Adhesion of the Probiotic Strain Escherichia coli Nissle to the Fungal Pathogen Candida albicans
Source: ACS Synth Biol. 2024 Sep 12;13(12):4027–39. doi: 10.1021/acssynbio.4c00466 (PMC11669158; doi:10.1021/acssynbio.4c00466)
Supplement: Supplementary file 1 — sb4c00466_si_001.pdf [file sb4c00466_si_001.pdf]

Supporting Information

Engineering adhesion of the probiotic strain *Escherichia coli* Nissle to the fungal pathogen *Candida albicans*

Alexandre Chamas[1,2,†,*], Carl-Magnus Svensson[3], Carla Maneira[1,4,5], Marta Sporniak[1], Marc Thilo Figge[2,3,4] and Gerald Lackner[1,2,5]

[1]Junior Research Group Synthetic Microbiology, Leibniz-Institute for Natural Product Research and Infection Biology, 07745 Jena, Germany.

[2]Cluster of Excellence Balance of the Microverse, Friedrich Schiller University Jena, 07743 Jena, Germany.

[3]Applied Systems Biology, Leibniz-Institute for Natural Product Research and Infection Biology, 07745 Jena, Germany.

[4]Institute of Microbiology, Faculty of Biological Sciences, Friedrich-Schiller University Jena, 07743 Jena, Germany.

[5]Chair of Biochemistry of Microorganisms, Faculty of Life Sciences: Food Nutrition and Health, University of Bayreuth, 95447 Bayreuth, Germany.

[†]current address: Department of Microbial Pathogenicity Mechanisms, Leibniz Institute for Natural Product Research and Infection Biology, 07745 Jena, Germany.

***To whom correspondence should be addressed**: alexandre.chamas@leibniz-hki.de

Supplementary tables and figures

Table S1: List of genetic parts utilized in this study.

genetic part	sequence
BBa_J23100 promoter	TTGACGGCTAGCTCAGTCCTAGGTACAGTGCT<br>AGC
BBa_J23114 promoter	TTTATGGCTAGCTCAGTCCTAGGTACAATGCT<br>AGC
AIDA signal peptide	AATAAGGCCTACAGTATCATTTGGAGCCACTC<br>CAGACAGGCCTGGATTGTGGCCTCAGAGTTAG<br>CCAGAGGACATGGTTTTGTCCTTGCAAAAAAT<br>ACACTGCTGGTATTGGCGGTTGTTTCCACAAT<br>CGGAAATGCATTTGCA
FLAG tag	GACTACAAAGACGATGACGACAAG
native linker sequence	ATCATTCTGAACCCGACTAAGGAATCTGCC
non-cleavable linker sequence	ATCATTCTGAACCCGACTAAGGAACTGACC
AIDA-C translocator region	GGTAATACTCTTACCGTGTCAAATTATACTGG<br>GACACCGGGAAGTGTTATTTCTCTTGGTGGTG<br>TGCTTGAAGGAGATAATTCACTTACGGACCGT<br>CTGGTGGTGAAAGGTAATACCTCTGGTCAAAG<br>TGACATCGTTTATGTCAATGAAGATGGCAGTG<br>GTGGTCAGACGAGAGATGGTATTAATATTATT<br>TCTGTAGAGGGAAATTCTGATGCAGAATTCTC<br>TCTGAAGAACCGCGTAGTTGCCGGAGCTTATG<br>ATTACACACTGCAGAAAGGAAACGAGAGTGG<br>GACAGATAATAAGGGATGGTATTTAACCAGTC<br>ATCTTCCCACATCTGATACCCGGCAATACAGA<br>CCGGAGAACGGAAGTTATGCTACCAATATGGC<br>ACTGGCTAACTCACTGTTCCTCATGGATTTGA<br>ATGAGCGTAAGCAATTCAGGGCCATGAGTGAT<br>AATACACAGCCTGAGTCTGCATCCGTGTGGAT<br>GAAGATCACTGGAGGAATAAGCTCTGGTAAG<br>CTGAATGACGGGCAAAATAAAACAACAACCA<br>ATCAGTTTATCAATCAGCTCGGGGGGGATATT<br>TATAAATTCCATGCTGAACAACTGGGTGATTT<br>TACCTTAGGGATTATGGGAGGATACGCGAATG<br>CAAAAGGTAAAACGATAAATTACACGAGCAA<br>CAAAGCTGCCAGAAACACACTGGATGGTTATT<br>CTGTCGGGGTATACGGTACGTGGTATCAGAAT<br>GGGGAAAATGCAACAGGGCTCTTTGCTGAAAC<br>TTGGATGCAATATAACTGGTTTAATGCATCAG<br>TGAAAGGTGACGGACTGGAAGAAGAAAAATA<br>TAATCTGAATGGTTTAACCGCTTCTGCAGGTG<br>GGGGATATAACCTGAATGTGCACACATGGACA<br>TCACCTGAAGGAATAACAGGTGAATTCTGGTT<br>ACAGCCTCATTTGCAGGCTGTCTGGATGGGGG<br>TTACACCGGATACACATCAGGAGGATAACGG<br>AACGGTGGTGCAGGGAGCAGGGAAAAATAAT<br>ATTCAGACAAAAGCAGGTATTCGTGCATCCTG<br>GAAGGTGAAAAGCACCCTGGATAAGGATACC

	GGGCGGAGGTTCCGTCCGTATATAGAGGCAAA<br>CTGGATCCATAACACTCATGAATTTGGTGTTA<br>AAATGAGTGATGACAGCCAGTTGTTGTCAGGT<br>AGCCGAAATCAGGGAGAGATAAAGACAGGTA<br>TTGAAGGGGTGATTACTCAAAACTTGTCAGTG<br>AATGGCGGAGTCGCATATCAGGCAGGAGGTC<br>ACGGGAGCAATGCCATCTCCGGAGCACTGGG<br>GATAAAATACAGCTTC
actinohivin CBD	GCGAGCGTTACCATCCGTAACGCACAGACCGG<br>CCGCCTCCTGGACTCTAACTATAACGGTAACG<br>TTTACACCCTGCCAGCGAACGGTGGCAACTAC<br>CAGCGTTGGACTGGTCCGGGTGACGGTACTGT<br>GCGCAACGCCCAGACTGGCCGTTGCCTGGATA<br>GCAACTATGACGGTGCTGTGTACACTCTGCCG<br>TGCAACGGCGGTTCCTACCAGAAATGGCTGTT<br>CTATTCTAATGGCTATATCCAAAACGTCGAAA<br>CTGGCCGTGTTCTGGATAGCAACTATAACGGT<br>AACGTGTATACCCTGCCGGCCAACGGCGGTAA<br>CTATCAGAAGTGGTATACCGGC
chi92 CBD	CATCCAGCATGGAGTGCAGGTACTGTGTACAA<br>CACGAATGACAAAGTATCCCACAAACAACTG<br>GTGTGGCAGGCCAAGTATTGGACCCAGGGCA<br>ATGAACCGAGCCGTACGGCCGATCAGTGGAA<br>ACTGGTTTCGCAGGTGCAATTAGGTTGGGATG<br>CGGGAGTGGTCTATAACGGCGGTGACGTAACC<br>TCTCATAACGGGCGCAAGTGGAAAGCTCAGTA<br>CTGGACAAAAGGCGATGAGCCGGGGAAAGCT<br>GCCGTCTGGGTTGATCAAGGCGCGGCGTCATG<br>CAAT
dectin-2 CBD	CGTCGCTTATATGAACTTCACACATACCATTCC<br>AGTCTCACCTGCTTCAGTGAAGGTACTATGGT<br>ATCCGAAAAAATGTGGGGATGCTGCCCGAATC<br>ATTGGAAATCATTTGGCTCCAGCTGCTACTTG<br>ATTTCTACCAAGGAGAACTTTTGGAGCACGAG<br>CGAGCAGAACTGTGTCCAGATGGGGGCCCATC<br>TGGTGGTGATCAATACTGAAGCGGAGCAGAAT<br>TTCATCACCCAGCAGCTGAACGAAAGCTTATC<br>TTATTTTCTGGGTCTGTCGGATCCACAAGGTA<br>ATGGCAAATGGCAATGGATCGACGATACGCC<br>GTTTAGTCAAAACGTCCGCTTCTGGCACCCCC<br>ATGAACCGAACCTACCGGAAGAGCGGTGTGTT<br>TCAATAGTGTACTGGAATCCTTCGAAATGGGG<br>CTGGAATGATGTTTTTTGTGACAGCAAACACA<br>ACTCGATTTGTGAAATGAAGAAAATTTATCTG
SP1 CBD	ATCAAAATTGAGTTCACTAGCCGTGAAGTACC<br>GTGGAACTCTAAGCTGGACGGCTACCTGGATG<br>ACGGTGCGACGCGCCTGTTTGCGTACCAGCAA<br>AACCACCCGGTGGCGGCCGTTCTGTCTACCCG<br>TATCATGTATGGTCCGATTAGCCGTCAGATCG<br>CCCGTGCGGACGAAGCACTGCATACCTTTTCT<br>AACCCACTGATCCTGGTGGCTGACAAAAGTC

	TCTGGTACCGGTTGAAAAGGGCACCTTCATGC TGGAAGGTAATCAATGCGGTGTTGAAGGCAA A
SP2 CBD	GCTAAAGCTGAAAGCACTTCCCGCGAAGTACC GGGCAACTCTAAGCTGGACGGTCGCCTCGACG ATGGTGCTACCCGTCTGTCTGCGGGTCAACAG AACCATCCAGTAGCGGCAGTGCTGAGCACCCG TGCCATGGGTGGCCCGAACAGCCGTCAGGACG CACGCGCGGATGAAGCGCTCCACACCGGCTCT AACCCGGGTAGCGGTGTTGCCGATAAGAAATC TCTCGTTCCGGTTGAAAAAGGCACTGCTATGC TGGAAGGTAACCAGTGTGGTGTTGAGGGTAAA
mKate2	ATGCTTACAAAAAAGAAGCTGAATGTTATATT AGTAAAGCTACGAGGAGGAACTACTATGGCTT CATCAGAACTTATCAAAGAAAACATGCACATG AAATTGTACATGGAAGGAACAGTAAATAATC ACCACTTTAAATGTACCTCAGAAGGAGAAGGA AAACCATATGAAGGTACTCAGACCATGCGTAT TAAGGCCGTTGAAGGTGGACCATTGCCTTTTG CCTTTGATATTCTTGCCACATCTTTTATGTACG GATCAAAAACTTTTATCAATCATACCCAAGGT ATCCCAGATTTCTTTAAACAGTCATTTCCTGAA GGATTTACATGGGAACGTGTCACAACTTATGA AGATGGTGGAGTATTGACAGCAACTCAAGATA CATCTCTGCAAGATGGTTGTCTTATCTACAAC GTAAAAATCCGTGGAGTTAATTTTCCATCTAA TGGTCCTGTTATGCAGAAAAAGACCCTTGGAT GGGAAGCATCTACGGAAACTTTATATCCTGCG GATGGCGGATTGGAAGGTCGTGCTGATATGGC TTTGAAACTTGTCGGTGGAGGTCACCTTATCT GTAATTTGAAGACCACATACCGTTCTAAAAAG CCAGCTAAAAATCTTAAGATGCCTGGTGTTTA CTACGTCGATCGTCGTTTAGAACGTATCAAAG AAGCAGATAAGGAAACTTATGTTGAGCAGCA CGAAGTAGCCGTCGCACGTTATTGTGATTTGC CTTCTAAATTGGGACACCGTTAA

Table S2: List of sequencing primers utilized in this study.

primer name	sequence	description
MS0_27	GTCGACATTTCCATCGCTGC	Confirmation of clbK-clbQ deletion fw
MSO_18	AATATGTTCCGGAGCAGCGG	Confirmation of clbK-clbQ deletion rv
AIDA seq_1	ATAACTTCCGTTCTCCGGTC	Confirmation of AIDA expression cassette cloning in pET-28 a (+) plasmid 1
AIDA seq_2	TATTAAAGAACGTGGACTCCA	Confirmation of AIDA expression cassette cloning in pET-28 a (+) plasmid 2

Table S3: Area values and other parameters determined by JIPipe for the example image of Fig.4.

parameter	values for Fig.4
hyphae area	5241948
total bacteria area	406800
overlap area = area of bacteria on hyphae	199157
percent hyphae covered with bacteria	3.80 %
percent bacteria area on hyphae	48.96 %

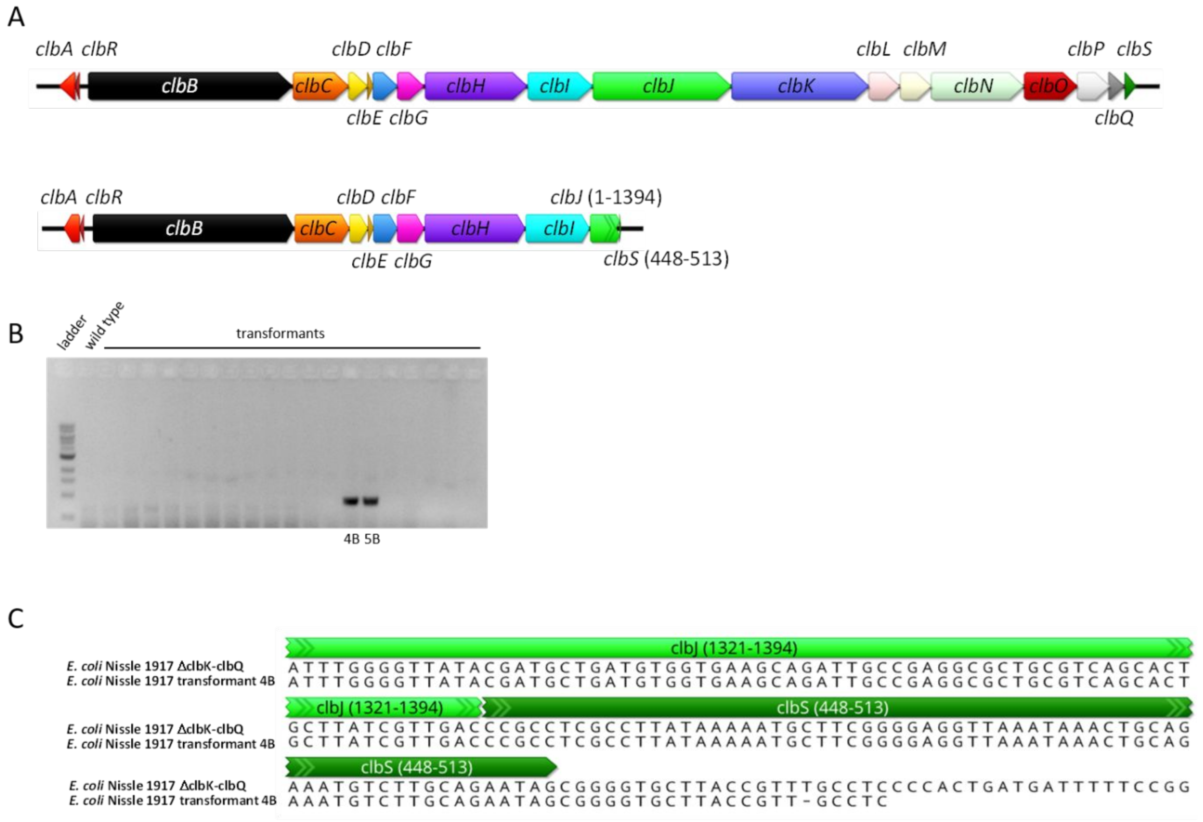

Fig. S1: (A) Schematic representation of the wild-type colibactin biosynthetic gene cluster (top) and the same cluster after No-Scar mediated deletion in mutant *E. coli* Nissle 1917 *ΔclbK-clbQ* (bottom). (B) Agarose gel confirming the deletion of part of the colibactin synthesis cluster. The genomic DNA of the wild type and of several transformants after No-Scar mediated deletion was extracted and submitted to a PCR with MSO_27 and MSO_18 primers. PCR mixtures were loaded on a 2 % agarose gel together with a 1 kb DNA ladder (New England Biolabs, Ipswich, USA). A correct deletion shows a PCR band of 840 bp, which is the case for transformants 4B and 5B. (C) Sequence alignment of clone 4B after No-Scar mediated deletion of genes *clbK* to *clbQ* with the *in silico* predicted genome sequence.

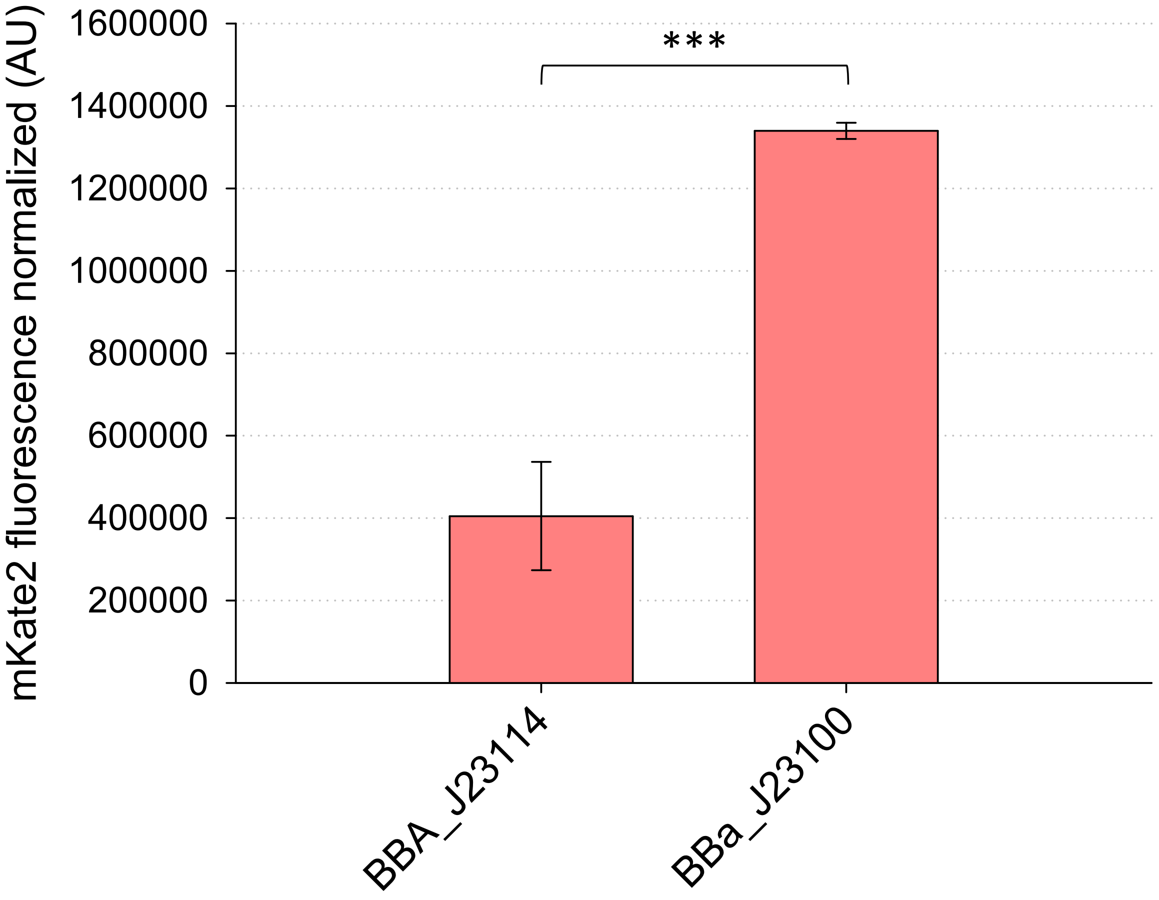

Fig. S2: Normalized mKate2 fluorescence for EcN strain expressing the BBa_J23114 or BBa_J23100 promoter upstream the mKate2 protein. Error bars represent the standard deviation of three biological replicates. Statistical test performed was a Student's t-test (two-tailed, P-value = 0.000260)

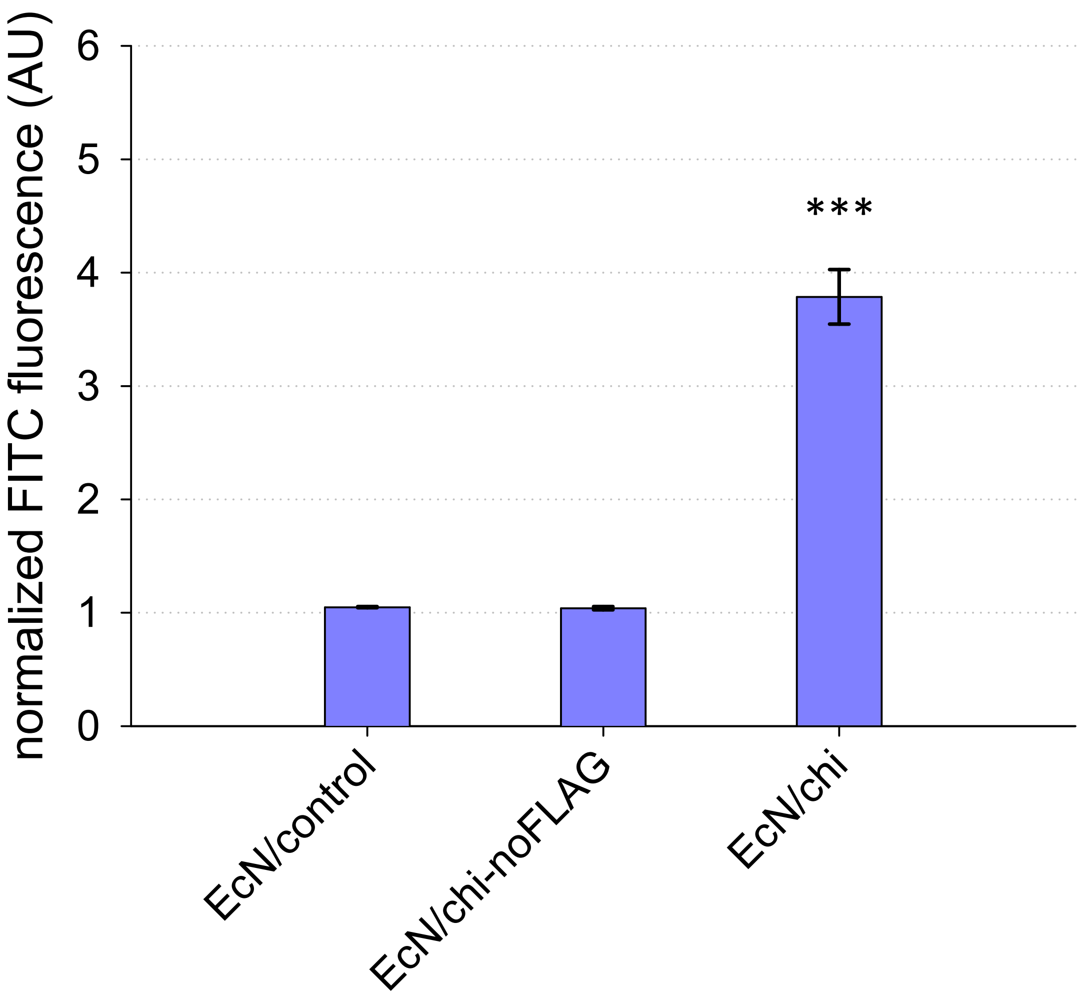

Fig. S3: FITC fluorescence of the indicated strains after incubation with an anti-FLAG antibody conjugated with FITC divided by the fluorescence of the same strains without antibody incubation. Error bars represent the standard deviation of three technical replicates. Statistical test performed was a one-way ANOVA with multiple comparisons versus EcN/control (Holm-Sidak method): *: $p<0.05$, **: $p<0.01$, ***: $p<0.001$.

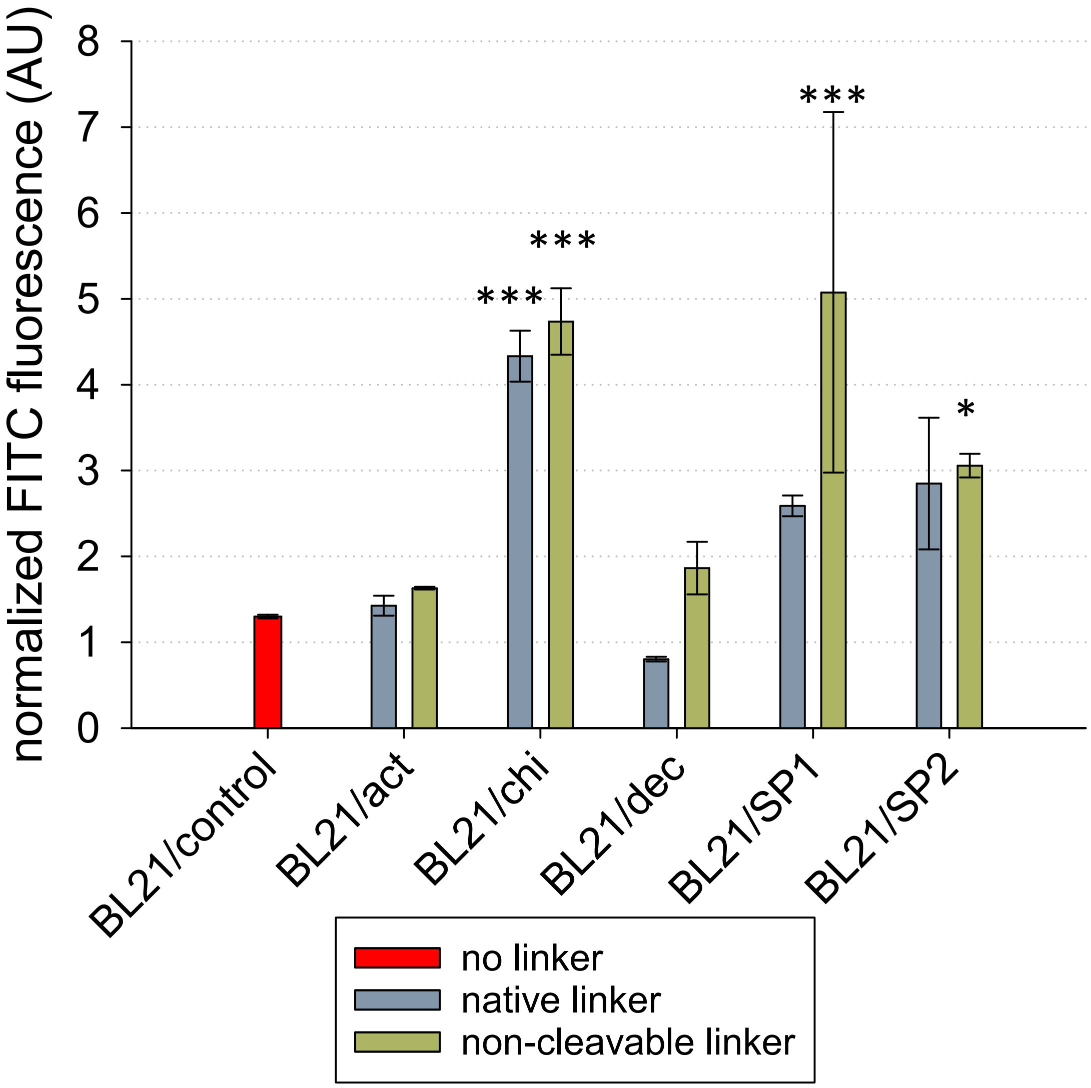

Fig. S4: FITC fluorescence of the indicated B21 strains after incubation with an anti-FLAG antibody conjugated with FITC divided by the fluorescence of the same strains without antibody incubation. Error bars represent the standard deviation of three technical replicates. Statistical test performed was a one-way ANOVA with multiple comparisons versus EcN/control (Holm-Sidak method): *: $p<0.05$, **: $p<0.01$, ***: $p<0.001$.

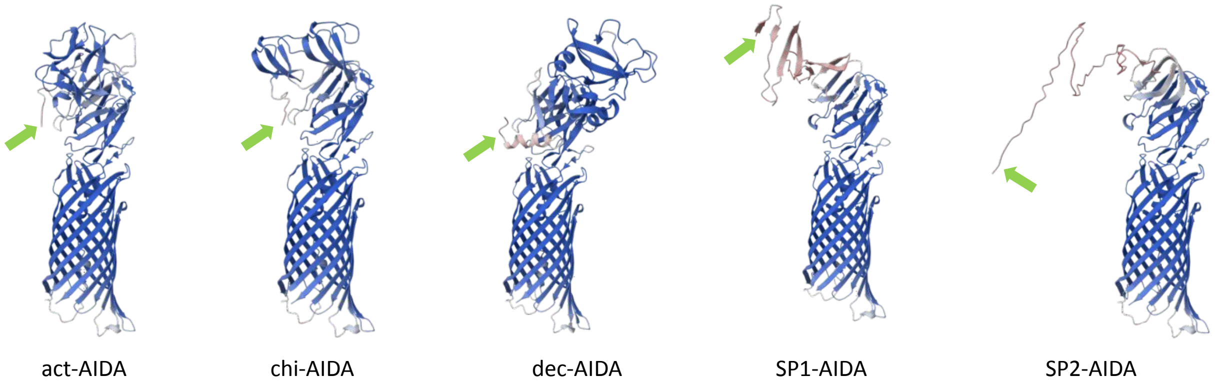

Fig. S5: Predicted protein structure of the indicated AIDA fusion proteins. Predictions were obtained with Alphafold2 and visualized with Mol* viewer*(51)*. The green arrow indicates the N-terminus.

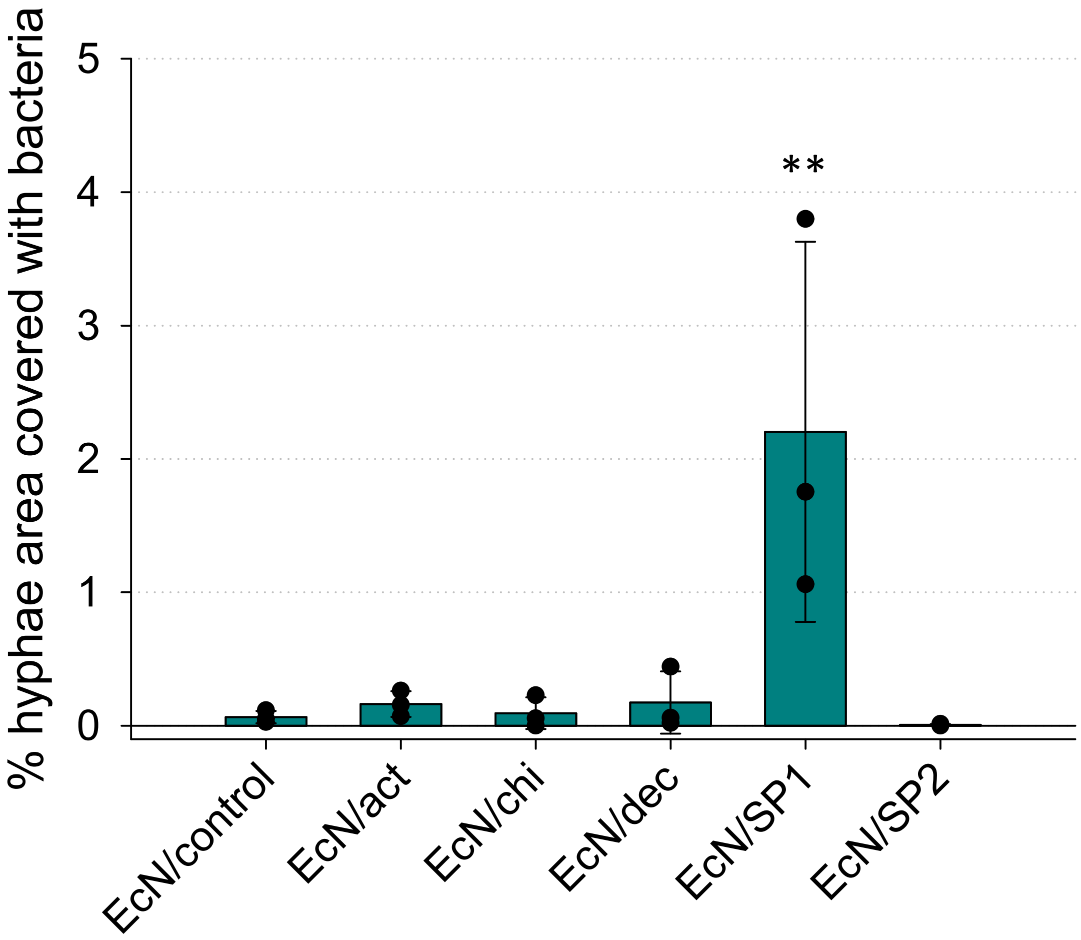

Fig. S6: Percent hyphae area covered with bacteria after hyphae-binding assay for the indicated strains after incubation for 4h. Dots show the values of nine replicates and bars show their mean with error bars representing the standard deviation. Statistical test performed was a one-way ANOVA with multiple comparisons versus EcN/control (Holm-Sidak method): *: $p<0.05$, **: $p<0.01$, ***: $p<0.001$.

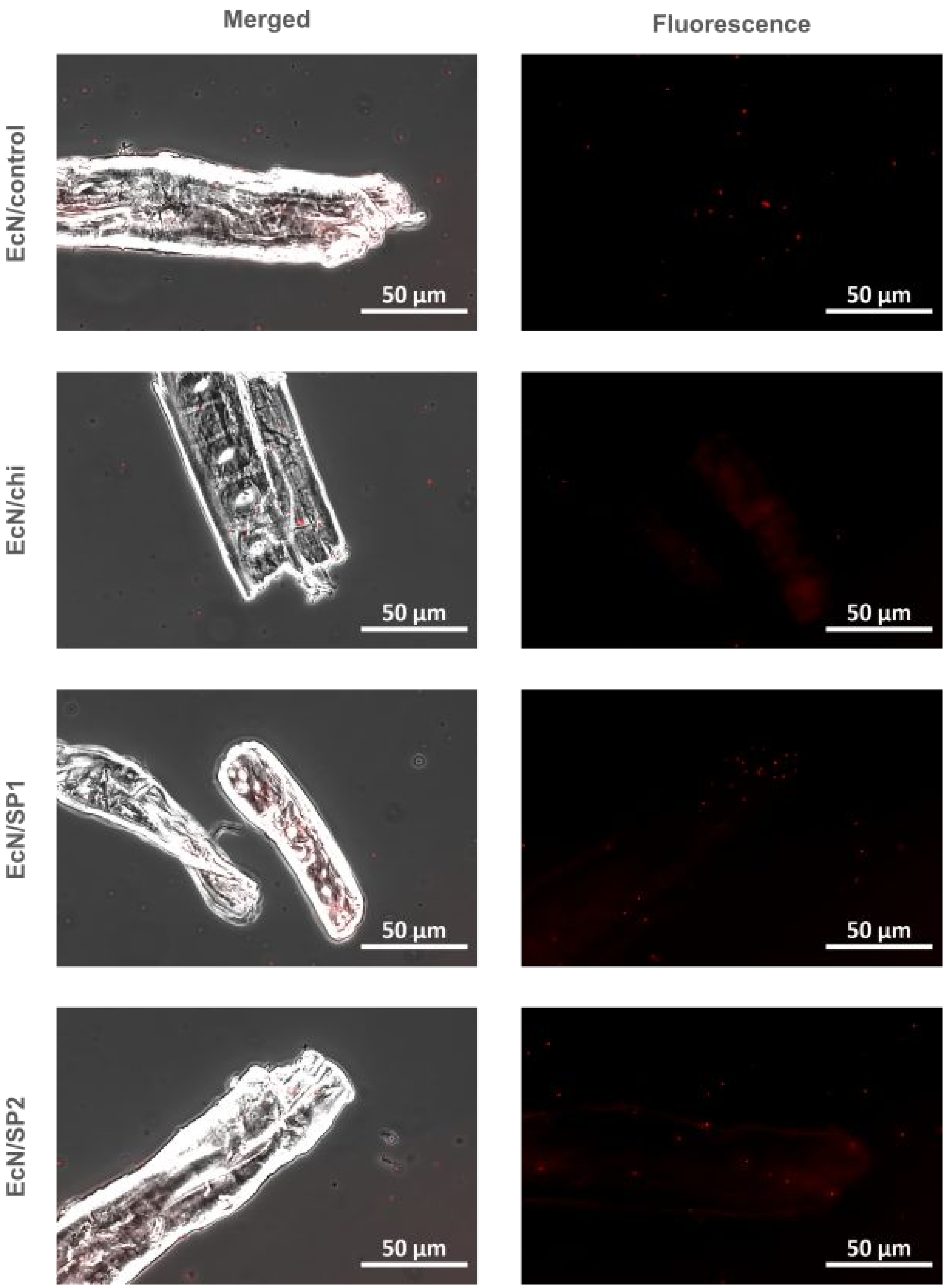

Fig. S7: Representative microscope images after incubation of the indicated strains with cellulose particles. Images are a merge of the brightfield and red fluorescence channel (left) or only the red fluorescence channel (right).

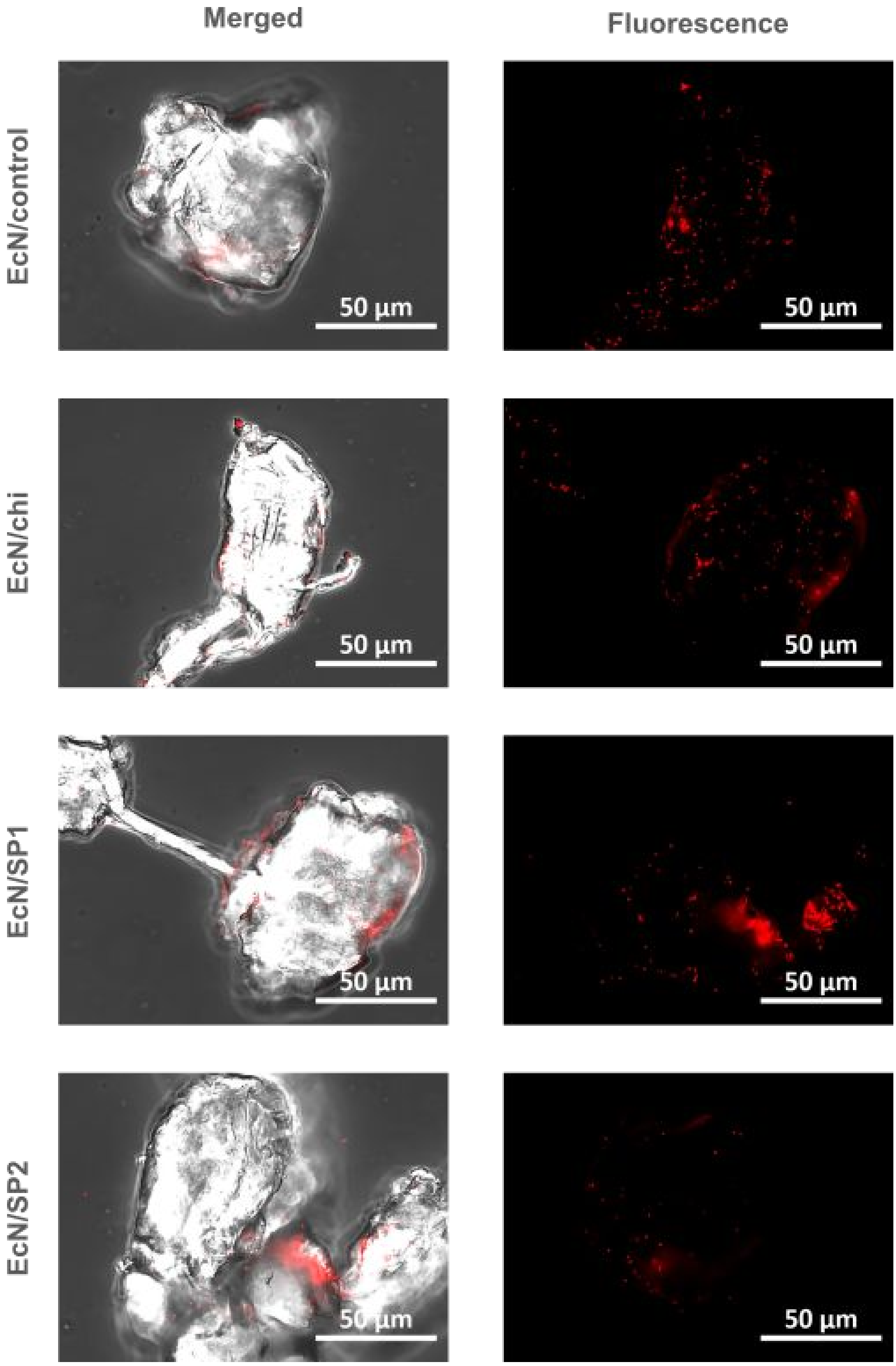

Fig. S8: Representative microscope images after incubation of the indicated strains with PET particles. Images presented are a merge of the brightfield and red fluorescence channel (left) or only the red fluorescence channel (right).
